# Interactions within the MHC contribute to the genetic architecture of celiac disease

**DOI:** 10.1371/journal.pone.0172826

**Published:** 2017-03-10

**Authors:** Benjamin Goudey, Gad Abraham, Eder Kikianty, Qiao Wang, Dave Rawlinson, Fan Shi, Izhak Haviv, Linda Stern, Adam Kowalczyk, Michael Inouye

**Affiliations:** 1 NICTA Victoria Research Lab, The University of Melbourne, Parkville, Victoria, Australia; 2 Centre for Epidemiology and Biostatistics, The University of Melbourne, Parkville, Victoria, Australia; 3 Department of Computing and Information Systems, The University of Melbourne, Parkville, Victoria, Australia; 4 IBM Research, Australia, Level 5, Carlton, Victoria, Australia; 5 Centre for Systems Genomics, The University of Melbourne, Parkville, Victoria, Australia; 6 School of BioSciences, The University of Melbourne, Parkville, Victoria, Australia; 7 Department of Pathology, The University of Melbourne, Parkville, Victoria, Australia; 8 Department of Mathematics, University of Johannesburg, Auckland Park, South Africa; 9 Faculty of Medicine, Bar Ilan University, Safed, Israel; 10 Center for Neural Engineering, The University of Melbourne, Parkville, Victoria, Australia; Baylor College of Medicine, UNITED STATES

## Abstract

Interaction analysis of GWAS can detect signal that would be ignored by single variant analysis, yet few robust interactions in humans have been detected. Recent work has highlighted interactions in the MHC region between known HLA risk haplotypes for various autoimmune diseases. To better understand the genetic interactions underlying celiac disease (CD), we have conducted exhaustive genome-wide scans for pairwise interactions in five independent CD case-control studies, using a rapid model-free approach to examine over 500 billion SNP pairs in total. We found 14 independent interaction signals within the MHC region that achieved stringent replication criteria across multiple studies and were independent of known CD risk HLA haplotypes. The strongest independent CD interaction signal corresponded to genes in the HLA class III region, in particular *PRRC2A* and *GPANK1/C6orf47*, which are known to contain variants for non-Hodgkin's lymphoma and early menopause, co-morbidities of celiac disease. Replicable evidence for statistical interaction outside the MHC was not observed. Both within and between European populations, we observed striking consistency of two-locus models and model distribution. Within the UK population, models of CD based on both interactions and additive single-SNP effects increased explained CD variance by approximately 1% over those of single SNPs. The interactions signal detected across the five cohorts indicates the presence of novel associations in the MHC region that cannot be detected using additive models. Our findings have implications for the determination of genetic architecture and, by extension, the use of human genetics for validation of therapeutic targets.

## Introduction

The limited success of genome-wide association studies (GWAS) to identify common variants that substantially explain the heritability of many complex human diseases and traits has led researchers to explore other potential sources of heritability (in the wide sense), including the low/rare allele frequency spectrum as well as interactions between genetic variants [1, 2]. Many studies are now leveraging high-throughput sequencing with initial findings beginning to elucidate the effects of low frequency alleles [3–6]. However, the characterization of statistical interactions between loci in complex human disease has been limited, despite the availability of a multitude of statistical approaches for interaction analysis of GWAS [7–13]. Large-scale systematic research into genetic interactions has been hampered by several computational and statistical challenges mainly stemming from the huge number of variables that need to be considered in the analysis (>100 billion pairs for even a small SNP array), the subsequent stringent statistical corrections necessary to avoid being swamped by large number of false positive results, and the requirement of large sample size in order to achieve adequate statistical power.

Despite these difficulties, several studies have begun to provide evidence for interactions between SNPs in several human diseases, including psoriasis [14], multiple sclerosis [15], Behçet’s disease [16], type 1 diabetes [17], Crohn's disease [18], bipolar disorder [11] and ankylosing spondylitis [19], as well as complex traits such as serum uric acid levels [20] and the expression levels of multiple genes in human peripheral blood [21]. Recent work has demonstrated the presence of non-additive interactions in the HLA region between known risk haplotypes for a number of autoimmune diseases [22]. Pairwise examination of known risk haplotypes for type 1 diabetes revealed 11 significant statistical interactions with evidence that these signals are caused by the position of specific amino acids that play a role in antigen presentation [23]. In celiac disease (CD), these interactions improve explanation of phenotypic variance beyond that of purely additive models and show strong improvements in predictive performance [22]. Importantly, these studies identify the HLA and wider MHC as a region of strong *a priori* evidence for non-additive interactions in autoimmune diseases. The success of these studies begs the question of whether further interactions can be detected using an agnostic “bottom-up” approach that examines all variants rather than a “top-down” approach focussing only on known risk loci and haplotypes.

While the studies listed above indicate that interaction analysis plays an important role in many conditions, the limited evidence for interactions detected from GWAS means that our characterisation of such joint effects between SNPs remains limited, especially when compared to our understanding of associations observed from individual risk variants. Several key questions remain including how wide-ranging interactions between variants are, how well interacting pairs replicate in other datasets, how the discovered interactions can be characterized in terms of previously hypothesized two-locus models [24, 25], whether it is possible to detect interaction signal in the presence of strong marginal signals [26, 27] and how much (if at all) interaction signals contributes to disease heritability [28].

Given these questions and motivated by the recent findings of interactions between HLA haplotypes in CD and other autoimmune diseases [22, 23, 29], we sought to detect and characterize specific SNP-SNP interactions, using CD as a basis for our analysis. CD is a complex human disease characterized by an autoimmune response to dietary gluten. CD has a strong genetic component largely concentrated in the MHC region, due to its dependence on the HLA-DQ2/DQ8 heterodimers encoded by the HLA class II genes *HLA-DQA1* and *HLA-DQB1* [30]. The genetic basis of CD in terms of individual SNP associations has been well characterized in several GWAS [31–37], including the additional albeit smaller contribution of non-HLA variants to disease risk [38]. The success of GWAS for common variants in CD has recently been emphasized by the development of a genomic risk score that could prove relevant in the diagnostic pathway of CD [39]. Autoimmune diseases have so far yielded the most convincing evidence for interactions both at a biological and statistical level [22, 23, 40], potentially due to power considerations since these diseases usually tend to depend on common variants of moderate to large effect within the MHC. Moreover, the effect of balancing selection, strong linkage disequilibrium and known long-range haplotypes that typify the MHC have long been thought to be indicative of epistatic interactions[41]. Given these findings in conjunction with recent observations that rare coding variants may play a negligible role in common autoimmune diseases [3], CD is a model disease in which to study interactions. Given the strong relationships observed between risk haplotypes, we hypothesised that further interactions in CD may be discovered amongst unassociated variants that could be discovered by examining all possible pairwise combinations of SNPs.

Here, we present a large-scale exhaustive study of pairwise interaction in celiac disease. Leveraging Genome-Wide Interaction Search (GWIS), a highly efficient approach for interaction analysis [42], we conduct genome-wide scans for all interacting pairs across five separate CD case/control datasets of European descent, finding thousands of statistically significant pairs despite stringent multiple testing corrections. Next, we show a high degree of concordance of these interactions across the datasets, demonstrating that they are highly robust and replicable. Given the complex LD and known risk haplotypes, we demonstrated that detected signals are novel, i.e. independent of known risk variants, and distil these into 20 independent interaction signals. Further, we characterize the common two-locus models found and compare them to previously proposed theoretical models. Finally, we examine whether interacting pairs add more predictive power and explain more disease variation than additive effects of single SNPs.

## Results

Datasets are summarized in **Table 1**, these include five independent, previously published GWAS datasets of CD with individuals genotyped from four different European ethnicities: United Kingdom (UK1 and UK2), Finland (FIN), The Netherlands (NL) and Italy (IT) [32, 33]. To limit the impact of genotyping error and other sources of non-biological variation, we implemented three stages of validation and quality control (QC): (i) standard QC within each dataset, (ii) independent exhaustive interaction scans within each of the five datasets, and (iii) derivation of a validated list of interactions based on UK1. The study workflow is shown in **S1 Fig**.

**Table 1:** Summary of datasets used in this study.

			Celiac cases		Controls		
		SNPs^a^	Samples^a^	Platform^b^	Samples^a^	Platform^b^	Reference
UK1	UK	301,546	763	Illumina Hap300v1-1	1420	Illumina Hap550	[30]
UK2	UK	515,413	1826	Illumina670-Quad	3777	Illumina 1.2M-Duo	[31]
FIN	Finland	513,952	647	Illumina670-Quad	1829	Illumina 610-Quad	[31]
NL	Netherlands	515,169	803	Illumina670-Quad	846	Illumina 670-Quad	[31]
IT	Italy	515,641	497	Illumina670-Quad	543	Illumina 670-Quad	[31]
Overlapping SNPs		290,277					

a. The number of samples/SNPs is reported after quality control procedures were applied.

b. All platforms contain a common set of Hap300 markers; the Hap550 and 610-Quad contain a common set of Hap550 markers.

### Exhaustive interaction scans and replication

For each dataset, we implemented stringent sample and SNP level quality control (**Methods**), and then conducted an exhaustive analysis of all possible SNP pairs using the GWIS methodology [42]. Each pair was tested using the Gain in Sensitivity and Specificity (GSS) statistic, which determines whether a pair of SNPs in combination provides significantly more discrimination of cases and controls than either SNP individually (**Methods**). Forty-five billion pairs were evaluated in the UK1 study (Illumina Hap300/Hap550) and 133 billion SNP pairs were evaluated in each of the four remaining cohorts (Illumina 670Quad and/or 1.2M-DuoCustom). Given this multiple testing burden, we adopted stringent Bonferroni-corrected significance levels of *P* = 1.1 x 10^−12^ for the UK1 and *P* = 3.75 × 10^−13^ for the remaining datasets. Examination of the distribution of observed GSS p-values relative to the uniform p-value distribution showed some deviation (**S2 Fig**), indicating the GSS statistic is overly liberal for p-values >10^−5^ yet overly conservative for p-values <10^−5^. We therefore used a permutation-based approach to adjust the observed p-values in a manner analogous to the widely used genomic control method (**Methods**). The resulting adjusted p-value distribution showed no test statistic inflation (**S2 Fig**).

To further ensure that the downstream results were robust to technological artefact and population stratification, we took two additional steps: (a) utilizing the raw genotype intensity data available for UK1 for independent cluster plot inspection of 696 SNPs comprising candidate interacting pairs, and (b) replicating the interactions of the SNPs passing cluster plot inspection, where replication is defined as a SNP pair exhibiting Bonferroni-adjusted significance both in UK1 and in at least one additional study. Using these criteria, we found that 5,454 SNP pairs (comprising 581 unique SNPs) from the UK1 dataset passed both (a) and (b) above. We denote these pairs as 'validated interaction pairs' (VIPs) below. The full list of VIPs is given in **S1 Dataset**. Notably, all VIPs fulfilling these robustness criteria were within the MHC.

**Fig 1:**
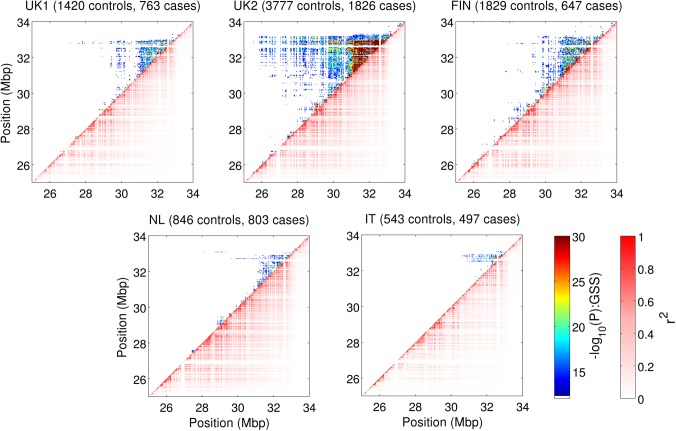
Interactions and LD patterns within the extended MHC region. SNP pairs within 30KB of each other are shown as a single point on each heatmap. The colour of each point in the upper left half of the graph represents the most significant -log_10_(P-value) returned by the GSS statistic for SNPs pairs within each point. The adjusted -log_10_(P-value) is capped at 30 to increase contrast of lower values. The bottom right half of the graph shows the maximum r^2^ obtained for any SNP pair within a given 30Kb block, demonstrating the strong LD patterns known to exist within this region.

More than 134,000 unique pairs achieved Bonferroni-adjusted significance across all five studies, with the vast majority lying within the extended MHC region of chr 6 (**Fig 1** and **Table 2**). Of the 35 pairs outside the MHC that were significant in at least one study, none passed Bonferroni-adjusted significance in at least one other study and were thus deemed not replicated. As expected, the number and significance of interactions increased with sample size. Interestingly, some of the strongest interactions tended to be in close proximity though few SNPs were in LD with only 12% of pairs having *r*^2^ >0.1 and 25% having an absolute D’ > 0.35. The number of pairs found at differing levels LD thresholds can be observed in **S3 Fig**. Analysis of the relationship between the GSS significance and LD shows little relationship, showing Spearman’s correlation of 0.17 (**S4 Fig**). The heatmaps in **Fig 1** also showed that interactions were widely distributed with distances of >1Mb common between interacting loci. While interactions were consistently located in and around HLA class II genes, further examination of the VIPs found that many of the strongest pairs were in HLA class III loci, >1Mb upstream of *HLA-DQA1* and *HLA-DQB1* (**S5 Fig**).

**Table 2:** Summary of the number of significant SNP pairs detected and of those that also replicate in at least one cohort.

Dataset	Two SNPs inside MHC	One SNP inside MHC	Both SNPs outside MHC
UK1	5,930 (5,454)	0 (0)	1 (0)
UK2	100,863 (30,351)	0 (0)	23 (0)
FIN	24,080 (8,058)	0 (0)	5 (0)
NL	2,505 (819)	0 (0)	0 (0)
IT	1,006 (397)	0 (0)	6 (0)
Unique pairs	134,384 (45,079)	0 (0)	35 (0)

For each dataset, we show the number of SNP pairs detected and number that appear as significant in at least one other cohort (in brackets). These have been separated into pairs where both SNPs are inside the MHC region, pairs with one SNP inside the MHC region and one outside and pairs where both SNPs are outside the MHC region.

**Fig 2:**
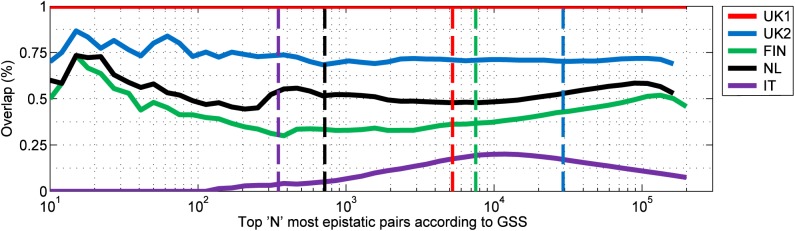
Replication of interacting SNP pairs between populations. Overlap of significant pairs as a percentage between UK1 and remaining cohorts in order of decreasing GSS significance. Vertical dotted lines indicate the Bonferroni-adjusted significance for each study.

The extent of replication of the detected interactions was apparent from the high degree of similarity in the rankings when pairs were sorted by GSS significance (**Fig 2**), with the top 10,000 pairs exhibiting ~70–80% overlap between the UK1 and UK2 datasets and 40–60% overlap of the UK1 with the pairs found in the NL and FIN datasets. Such high degrees of overlap have essentially zero probability of occurring by chance. The pairs found in the IT dataset showed lower levels of consistency with those detected in the UK1 dataset but overall were still far more than expected by chance with ~30% overlap at ~30,000 pairs.

### Independence of validated interaction pairs and known HLA risk haplotypes

The large number of VIPs demonstrates that thousands of SNP pairs showing significant interaction effects can reliably be found in the HLA region. However, while the GSS test is designed to select VIPs exhibiting significant statistical interaction effects, the biological interpretation of these effects is not obvious. Given that recent work has shown that significant statistical interactions can be induced by haplotype structure [27, 43], it is important to demonstrate that detected interactions are independent of the known *HLA-DQA1/DQB1* risk haplotypes known to be instrumental in CD aetiology, especially given the VIPs’ co-localization within a region of complex linkage disequilibrium. Furthermore, as detected pairs may be correlated with each other, we also wish to estimate the number of independent interaction signals detected.

To determine whether VIPs were independent of known haplotypes we utilized a second filtering step in the form of a likelihood ratio test (LRT), testing whether adding a VIP to a logistic regression model of case/control status on the haplotypes increased model fit significantly, with the threshold for significance defined using a false discovery rate (FDR) of 5% (*P* value < 0.044) and including the top five principal components to control for population structure (**Methods**). Due to the extensive LD in the region, it is inevitable that some VIPs will be fully captured by HLA risk haplotypes; as a subsequent filter to the GSS test, the LRT will identify these as well as determine whether each VIP contains significantly more independent information on CD risk than the HLA risk haplotype alone. After keeping the VIPs that were independent of risk haplotypes, we used an LD pruning based approach based on Hill's *Q*-statistic, a normalized chi-squared statistic for multi-allelic loci [44], to estimate the number of haplotype-independent interaction pairs that were also independent of each other.

From the 5,454 VIPs, we found that 4,227 pairs (>77% of VIPs) were independent of the known CD risk alleles (DQB1*03:*02*, DQB1*03:*01*, DQB1*02:*02*, DQB1*02:*01*, DQA1*03:*01*, DQA1*0:*505*, DQA1*05:*01*, and DQA1*02:*01***)**. From the 4,227 VIPs independent of risk HLA haplotypes, LD pruning using a cutoff of Hill's *Q* = 0.3 identified 14 VIPs in UK1 which represent independent interaction signals (**Methods, Table 3,** significance per dataset is shown in **S1 Table**). Similar trends can be observed in other cohorts, with a high degree of signal overlap between datasets (**S2 Table**). Exploring the functional properties reveals over half of these SNPs have been previously indicated to be eQTLs, with many showing evidence that they disrupt transcription factor binding. Several of the SNPs detected have also been previously associated with autoimmune conditions including rheumatoid arthritis[45], multiple sclerosis[46] and IgA nephropathy[47, 48], providing evidence for cross-phenotypic, and potentially pleiotropic, effects.

**Table 3:** Independent validated interaction signals detected in UK1.

	SNP	RS	Chr	Position (bp)	Closest Gene ^a^	Distance to gene (bp) ^a^	eQTL ^b^	Evidence of TF binding ^c^	Previously reported ^d^
1	hg18.chr6:g.31701455G>A	rs2260000	6	31701455	SNORA38	-2489	-	Low	-
	hg18.chr6:g.31736712C>T	rs805262	6	31736712	C6orf47	-184	-	Low	-
2	hg18.chr6:g.32777745G>A	rs2647050	6	32777745	HLA-DQB1	-35301	-	Low	Emphysema imaging phenotypes
	hg18.chr6:g.32778934A>G	rs2856705	6	32778934	HLA-DQB1	-36490	-	-	-
3	hg18.chr6:g.29719410A>G	rs29232	6	29719410	GABBR1	-10519	-	-	Nasopharyngeal carcinoma
	hg18.chr6:g.29775252T>C	rs7776082	6	29775252	ZFP57	-22342	-	-	-
4	hg18.chr6:g.32685358G>A	rs660895	6	32685358	HLA-DRB1	-19767	GTEx/Regulome	High	Rheumatoid arthritis, IgA nephropathy
	hg18.chr6:g.32877641C>T	rs2219893	6	32877641	HLA-DOB	10876	-	Low	-
5	hg18.chr6:g.31192414T>C	rs1062470	6	31192414	CDSN	1571	GTEx	Low	-
	hg18.chr6:g.31317489G>A	rs3130712	6	31317489	HLA-C	27015	Regulome	High	-
6	hg18.chr6:g.32259421T>C	rs2070600	6	32259421	AGER	-656	-	Low	Pulmonary function, Emphysema imaging phenotypes
	hg18.chr6:g.32514320A>C	rs3129871	6	32514320	HLA-DRA	1276	-	Low	Multiple sclerosis
7	hg18.chr6:g.31429190T>C	rs2596501	6	31429190	HLA-B	431	GTEx/Regulome	High	-
	hg18.chr6:g.31886251C>T	rs2227956	6	31886251	HSPA1L	877	Regulome	High	-
8	hg18.chr6:g.32484449C>A	rs3763313	6	32484449	BTNL2	-1564	Regulome	High	HIV
	hg18.chr6:g.32871088G>A	rs2621377	6	32871088	HLA-DOB	17429	-	-	-
9	hg18.chr6:g.27520365A>G	rs7772160	6	27520365	ZNF184	134	GTEx	-	-
	hg18.chr6:g.27588896C>T	rs6918131	6	27588896	ZNF184	-40020	GTEx	Low	-
10	hg18.chr6:g.31630648A>G	rs6929796	6	31630648	NFKBIL1	-3937	GTEx	Low	-
	hg18.chr6:g.31644203T>C	rs2844484	6	31644203	LTA	3651	GTEx/Regulome	High	-
11	hg18.chr6:g.33147603C>T	rs9277341	6	33147603	HLA-DPA1	-1829	Regulome	High	-
	hg18.chr6:g.33191099G>A	rs1810472	6	33191099	HLA-DPB2	2829	Regulome	High	-
12	hg18.chr6:g.32919361G>A	rs6924102	6	32919361	PSMB8	-433	GTEx	Medium	
	hg18.chr6:g.32919607A>C	rs2071543	6	32919607	PSMB8	-187	GTEx	Low	IgA nephropathy
13	hg18.chr6:g.31572718T>C	rs3828903	6	31572718	MICB	2082	GTEx/Regulome	High	-
	hg18.chr6:g.32845485A>G	rs9368741	6	32845485	HLA-DQB2	-6177	-	Low	-
14	hg18.chr6:g.31002738T>C	rs2532934	6	31002738	VARS2	-524	Regulome	High	
	hg18.chr6:g.32517508G>A	rs3129882	6	32517508	HLA-DRA	1912	Regulome	High	Parkinson's disease, Systemic sclerosis

a. Closest gene and distance to that gene are derived from the UCSC RefGene annotation for HG18

b. eQTL column indicates whether a given SNP is an eQTL with evidence indicating that the SNP is reported as a significant eQTL in at least one tissue type in GTEx (gtexportal.org) or has been marked as being linked to gene expression in RegulomeDB (regulome.stanford.org). The genes reported as being regulated by these eQTLs are reported in **S3 Table.**

c. Level of evidence that a given SNP is likely to affect binding as described by RegulomeDB. For ease of interpretation, we have binned the RegulomeDB scores further into ‘high’ (1a-1f), ‘medium’ (2a-3), and ‘low’ (4–6) evidence categories.

d. Phenotype that has been previously associated with the given SNP as reported in the NHGRI-EBI GWAS Catalog.

We further demonstrated the independence of VIPs from known risk factor by employing a LRT evaluating whether the interaction effects for the VIPs holds using a logistic regression-based approach as well as conditioning on the five principal components to control for population structure, strong univariate associated SNPs and known celiac haplotypes, inferred using HIBAG [49] (**Methods**). A logistic-regression based test for interaction will differ from the model-free GSS-based test, as it will only detect interactions relative to the log-odds scale while GSS may detect interactions regardless of scale. Nevertheless, we find 521 VIPs (including 509 of the haplotype independent VIPs) are significant past Bonferroni correction (*P* < 9.12 × 10^−6^) using a meta-analysis based approach **(Methods, S6 Fig**) with many showing p-values below 10^−12^. We further validate that these results are robust to the large effect sizes observed within the HLA region through simulations comparing the distribution of significance for interactions where one loci has a strong univariate effect size to interactions where both loci are simulated under the null hypothesis of no effect (**Methods, S7 Fig**). Both scenarios show minimal deviation from a purely uniform distribution, indicating, the use of a logistic regression based test for interaction is robust and unlikely to be highly skewed by loci of large effect. These results all indicate that despite a different definition of interaction and the potential loss of power due to overadjustment, many robust interaction signals remain highly significant.

### Empirical two-locus model distributions

Two-locus disease models are typically represented as a table of penetrance values with one penetrance value for each genotype combination [7] and provide insight into how disease risk is distributed. Such models are of interest due to their natural role in the inference of disease mechanism for two or more genes as well as inference of population specific, and therefore potentially evolutionary, effects thereof. Further, characterization of two-locus model frequencies also enables the development of more powerful statistical approaches that target frequent models. While **Table 3** shows the models for each of the interacting pairs chosen to represent the independent signals detected, we are interested in the overall model consistency and distribution across all cohorts and have therefore analysed two-locus models across all VIPs. Following the conventions of Li and Reich [24], we discretized the models for the VIPs to use fully-penetrant values where each genotype combination implies a complete susceptibility or protective effect on disease (**Methods**), simplifying the comparison of models between different SNP pairs.

**Fig 3:**
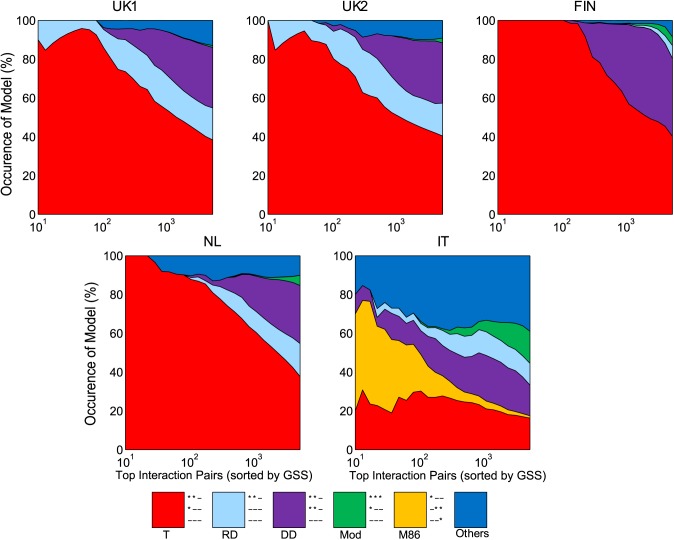
Variation in two-locus models within and between populations. Distribution of two-locus models for VIPs in different studies as increasingly less significant SNP pairs are examined. Different colours represent a different subset of two-locus models. The “other” group represents the remaining set of models. Models have been simplified using the rules provided in [24].

To establish model consistency, we first replicated the most frequent full penetrance VIP models in the other datasets (**Fig 3**). When considering the distribution of two-locus models we found striking consistency of the UK1 models with those from UK2 and the other Northern European populations (Finnish and Dutch). Only four models from the possible 50 classes [24] occurred with >5% frequency in the Northern European studies, and there was substantial variation in two-locus model as a function of the strength of the interaction. Amongst all VIPs in UK1, the four models corresponded to the threshold model (T; 38.3% frequency), jointly dominant-dominant model (DD; 31.1%), jointly recessive-dominant model (RD; 16.5%), and modifying effect model (Mod; 1.0%) [24, 50]. The DD and RD models are considered multiplicative, the Mod model is conditionally dominant (i.e., one variant behaves like a dominant model if the other variant takes a certain genotype), and the T model is recessive. The T model was the most frequent model, especially amongst the strongest pairs.

Interestingly, despite the consistency of MHC interactions, the VIPs showed noticeable differences in two-locus model distribution in the IT dataset. This was in contrast to the other Northern European populations but consistent with the different ranking in GSS significance (**Fig 2**). In the IT dataset, the distribution of models was altered such that there was a more even distribution. The four most frequent models were still the T model (16.2%), modifying effects (16.5%), DD (15.8%), and RD model (11.3%). However, we also observed that many of the strongest pairs within the IT dataset followed the M86 model, though M86 represented only a small proportion of models overall (1.2%). The other VIP models overall were relatively uniform amongst the remaining models.

The cause(s) of the differences in two-locus model distribution for the IT data are not clear, however it is unlikely due to sample size. While cryptic technical factors cannot be ruled out at this stage, we speculate that there may be population specific variation that follows the known North/South European genetic gradient [51]. Such variation in interaction analysis has been previously shown in the evolution of complex genetic systems [52], however evidence of such phenomena in human genetics has not, to our knowledge, been uncovered. While the differences in two-locus model for the IT population relative to Northern Europeans are notable, further studies specifically evaluating the consistency of two-locus model variation between populations (and potentially between diseases) are required to validate its evolutionary basis.

### Contribution of interacting pairs to celiac disease variance

We next sought to estimate the CD variance explained by the detected pairwise interactions and single SNPs. To do this, we utilized a multivariable model framework which accounted for all SNPs and/or VIPs at once. To assess the contribution of interacting loci to CD prediction and thus genetic variance explained, we employed L1-penalized linear support vector machines (SVM, see Methods), an approach which models all variables concurrently (single SNPs and/or pairs) and which has been previously shown to be particularly suited for maximizing predictive ability from SNPs in CD and other autoimmune diseases [39, 53]. We have previously shown that additive models of single SNPs explain substantially more CD variance than haplotype-based models [30], thus we employ only the former to estimate the gain in CD variance explained here.

We assessed CD variance explained by constructing three separate models: (a) genome-wide single SNPs only, (b) the VIPs only, and (c) a 'combined' model of both single SNPs and VIPs together. The models were evaluated in cross-validation on the UK1 dataset, and the best models in terms of Area Under the Curve (AUC) were then taken forward for external validation in the other four datasets without further modification.

In UK1 cross-validation, the combined models led to an increase of ~1.2% in explained CD variance, from 32.9% to 34.1% (respectively, AUC of 0.883 and 0.888) (**Table 4**). In external validation, the models based solely on VIPs had overall high predictive ability across all external validation datasets (AUC > 0.85), but slightly less than models based on single SNPs alone. The combined models yielded the highest externally validated AUC of all models, showing gains in AUC over single SNPs of +0.9% AUC in UK2 (Delong's 2-sided test *P* = 1.14×10^−6^). In the IT dataset, average gains were higher at +1.1% AUC yet marginally significant (*P* = 0.0535). In the FIN dataset AUC increased by +0.8% (*P* = 0.0351), however, in NL the increase was smaller (0.4%) and not statistically significant (*P* = 0.177).

**Table 4:** Disease variance explained by models with additive and interacting genetic effects.

		Single SNPs		Combined		Validated interaction pairs	
		Variance explained	AUC	Variance explained	AUC	Variance explained	AUC
**Cross validation**	UK1	0.329	0.883	0.341	0.888	0.339	0.887
**External validation**	UK2	0.269	0.855	0.288	0.864	0.267	0.854
	Finn	0.323	0.880	0.342	0.888	0.320	0.879
	IT	0.273	0.857	0.295	0.868	0.265	0.853
	NL	0.271	0.856	0.280	0.860	0.266	0.853

Predictive power and disease variance explained by single SNPs and VEPs in cross-validation and in external validation, using SparSNP models. Models were optimized on the UK1 dataset (n = 2183 samples) in cross-validation (290K SNPs), and tested without modification on the other datasets. The proportion of disease variance explained (on the liability scale) assumes a population prevalence of 1%. Two-sided DeLong significance tests for AUC of single SNPs+pairs difference from AUC of single SNPs: UK2 *P* = 1.14×10^−6^, FIN *P* = 0.0351, IT *P* = 0.0535, NL *P* = 0.1772.

Combining the UK1 and UK2 into a single dataset (N = 7,786 unrelated individuals) and retraining the models in cross-validation showed similar trends with the best models being the combined models (**S4 Table**). The combined models from the larger UK1+UK2 dataset also showed higher AUC in external validation than the models trained on UK1 data only, when validating the combined SNP + VIP models on the FIN and NL datasets (AUCs +1% and +0.9%; *P* = 0.00238 and *P* = 0.0032, respectively); however, performance on the IT dataset did not differ significantly (+0.8% AUC, *P* = 0.1037).

## Discussion

This study has shown the robust presence of interactions in celiac disease. The SNP pairs were mostly independent of HLA risk haplotypes for CD and strongly replicate across cohorts in terms of significance, ranking, and two-locus model. Many also remained significant under a regression-based definition of interaction. To our knowledge, this level of interaction signal strength, number of interacting SNP pairs, and degree of replication has not been previously shown in a complex human disease. We also performed a large-scale empirical characterization of the two-locus models underlying the interactions in CD, with the majority of the VIPs approximately following the threshold model, and a smaller number following dominant-dominant, dominant-recessive, and recessive-recessive models. Further, these patterns were found to be strongly consistent across most of the datasets.

Despite observations that interactions between SNPs within a locus are enriched for batch effects and poorly clustered genotype clouds [18], the stringent quality control and extensive replication in this study indicate that these SNPs represent novel signal rather than technical artefact. A large number of candidate SNP pairs that did not achieve Bonferroni significance criteria for replication still showed strong interaction effects associated with CD consistently across datasets, indicating that our estimates of the degree of interaction in CD may be conservative.

For validated interaction pairs, we found that much of the strongest signal was >1Mb upstream of the well-known *HLA-DQA1* and *HLA-DQB1* risk loci and suggested a potentially important contribution from HLA class III genes. Indeed the strongest interaction signal, which was independent of HLA risk haplotypes and other VIPs, was attributable to variants in *PRRC2A* and *GPANK1/C6orf47*. Given that individuals with celiac disease are at elevated risk of non-Hodgkin's lymphoma (NHL), it is intriguing that variants within *PRRC2A* are also associated with NHL [54]. However, for the top *PRRC2A* SNP for NHL (*rs3132453*, *hg18*.*chr6*:*g*.*31712023A>C*) we did not observe a validated interaction relationship nor linkage disequilibrium between *rs3132453*:*A>C* and the interacting *PRRC2A* SNP (*rs2260000*, *hg18*.*chr6*:*g*.*31701455G>A*), which was low in the HapMap2 CEU (*r*^2^ = 0.05). There is also evidence to suggest that women with celiac disease are at increased risk of early menopause [55, 56]. A recent genetic association study of menopausal age identified a missense variant within *PRRC2A* (*rs1046089*, *hg18*.*chr6*:*g*.*31710946T>C*) which was predicted as both structurally damaging for PRRC2A as well as an expression QTL for multiple genes [57]. In our study, the *PRRC2A* SNP *rs1046089*:*T>C* showed strong interaction effects with another proximal variant in *ABHD16A* as well as several other variants in the MHC despite low LD with the strongest *PRRC2A* interaction variant (*r*^2^ = 0.27). Overall, our findings indicate that, in addition to the known HLA risk haplotypes for CD, there is interaction between HLA class III loci, which may have implications for CD co-morbidity.

Despite the application of interaction analysis to GWAS becoming more widespread, interpretation of detected signals remains difficult. We have demonstrated that interactions detected in this work are not driven by technical artefact and are not tagging known risk factors using multiple conditional analyses and corroborating this with extensive replication. Nevertheless, this does not imply the statistical interactions are indicative of “functional epistasis” [52], the biological interaction of two loci, as some statistical interactions have been shown to tag local haplotype structure [27, 43]. Interestingly, models of only significantly associated interaction pairs explained nearly as much CD variance as additive models of all single SNPs. One interpretation of this could be that the haplotype signals in the MHC regions are tagged by both single SNPs and SNP pairs, leading to an overlap in detected signal, with pairs capturing some novel signal within the MHC while single SNP analysis also includes signal from outside this region. This observation adds to an increasing body of literature supporting the existence of shared information between additive and epistatic effects [21, 58]. While biological interpretation of detected interactions remains difficult, the interactions detected in this work and in several recent reports [22, 23] highlight that interaction analysis can detect novel signal which cannot be detected from standard additive analysis and can provide plausible hypotheses for follow-up investigation.

While the challenge of separating additive and non-additive signals does not affect studies in the genomic prediction of complex diseases [39], it implies that determining causal genetic signals of CD, and perhaps other autoimmune/inflammatory diseases with substantial HLA-based effects, is more difficult than previously thought. Determining the true genetic effects is central to the identification of the molecular products involved in pathogenesis, thus the usage of genetic data in the discovery and validation of therapeutic targets [59] would benefit from the exploration and potential resolution of the interaction and additive components of a given disease. While the increase in explainable variance from interaction signals in this study was low, candidate interactions discovered through pairwise analysis may still provide important therapeutic targets [60], regardless of whether these targets are interacting loci or previously unassociated haplotypes. Given this, and in conjunction with recent findings that support the evolutionary persistence of substantial non-additive effects [26], our findings should also stimulate debate around whether the usage of the principle of parsimony (Occam's Razor) is an adequate rationale for models of exclusively additive effects [28].

The limitations of the first generation GWAS approach to explain missing heritability has led to the development and application of more sophisticated approaches to resolve this problem, yet success has been elusive. Recent results suggest that rare variants add little to known heritability for a number of autoimmune diseases including celiac disease [3]. In contrast, an increasing body suggests that interactions play a strong role in such conditions [14, 15, 19, 22, 23], lending evidence to the hypothesis that strong, epistatic interactions exist within the HLA region[39]. Despite the difficulty in determining the biological mechanism at play, the use of interaction analysis may offer both additional explained (broad-sense) heritability as well as helping to identify novel associated regions, which can then be further explored. The predictive models of CD generated in this work demonstrate that interacting pairs explain disease variance significantly beyond that of purely additive models, revealing novel signal that will go otherwise undetected. These findings have implications for the genetic architecture of celiac disease, the incorporation of interactions into genetic models of complex disease, and the use of human genetics in therapeutic target validation.

## Methods

### Samples and quality control

We analyzed genotype data from previously published GWAS studies of Celiac disease (EGA accession EGAS00000000057). A range of quality-control measures were applied to all datasets to limit the impact of genotyping error. For all datasets, we removed non-autosomal SNPs, SNPs with MAF <5%, missingness >1% and those deviating from Hardy-Weinberg Equilibrium in controls with *P* < 5 x 10^−6^. Samples were removed if data missingness was >1%. Cryptic relatedness was also stringently assessed by examining all pairs of samples using identity-by-descent in PLINK, and removing one of the samples if $\hat{\pi }$ > 0.05. The cryptic relatedness filter removed 17 samples within the UK1 cohort that related to other UK1 samples, and 1208 samples from the UK2 cohort that were either related to other UK2 samples or UK1 samples. Dataset sizes in **Table 1** are reported after the quality control steps above. SNP pairs showing significant interaction effects were further assessed by manually inspecting the genotyping cluster plots of both SNPs in the UK1 cohort. Intensity data for the other studies was not available. Cluster plot inspection removed 115 SNPs with poor genotyping assays.

### Interaction analysis

The Gain in Sensitivity and Specificity (GSS) test was employed to detect interaction effects. The test has been presented in detail in [42] and is summarized in **S1 Text**. It is available at https://github.com/bwgoudey/gwis-stats.

### Odds ratio for interacting pairs

Analogously to odds ratios used for analyses of single SNPs, we define odds ratios for interaction pairs based on the GSS statistic

$$OR_{GSS}=\frac{\left(\pi_{\left\{1,HR\right\}})(\pi_{\left\{0,LR\right\}}\right)}{\left(\pi_{\left\{0,HR\right\}})(\pi_{\left\{1,LR\right\}}\right)},$$

where π_(i,j)_ denotes the proportion of samples with phenotype *i*, 1 for cases and 0 for controls, and carrying genotype combinations which are marked as *j* with *HR* (high risk) indicating genotypes which are associated by GSS with cases and *LR* (low risk) indicating genotypes which are associated with controls (analogous to MDR-style approaches in [12, 61]). By relying on the model-free GSS approach, this odds ratio can be seen as a measure of association for the combination of genotypes from a given SNP pair which has the strongest improvement over the pair’s SNPs.

### Representation of the two-locus models

We approximate the two-locus models for VIPs using two representations: balanced penetrance models and full penetrance models. Following Li and Reich [24] we employ the penetrance, that is, the probability of disease given the genotype, estimated from the data for each of the nine genotype combinations as (number of cases with combination) / (number of individuals with combination). Representing the two-locus model in terms of penetrance allows us to clearly see which genotype combinations contribute more to disease risk (or conversely, may be protective). We employ a standardization to ensure that the penetrance is comparable across datasets, termed *balanced sample penetrance*, and defined as

$$P_{balanced,v}=\frac{p_{1v}}{p_{1v}+p_{0v}},$$

where *p*_*iv*_ refers to the proportional frequency of genotype *v* in class *i*, where controls are 0 and cases are 1. The definition is easily extended to the case of pair of SNPs using the 3 × 3 = 9 possible genotype combinations from each SNP-pair.

For comparison of models, we employ a coarse-grain approach where these values are discretized into binary values, so called “fully penetrant” models, similar to Li and Reich [24]. Unlike Li and Reich, we do not swap the high and low risk status, as we are interested in distinguishing between protective and deleterious combinations. In addition, we do not swap risk status, therefore there will be 100 possible full-penetrance models. For rare genotype combinations we used a simple heuristic, denoting all cells with a frequency below 1% in both cases and controls as ‘low risk’. Experiments with this threshold revealed that altering this cut-off between 0% and 7% made little difference to the overall distribution of our models.

### Independence of interaction signals from known HLA risk haplotypes

CD strongly depends on specific heterodimers, most notably HLA-DQ2.2, HLA-DQ2.5, and HLA-DQ8, which are in encoded by haplotypes involving the *HLA-DQA1* and *HLA-DQB1* genes, with close to 100% of individuals with CD being positive for one of these molecules. To statistically impute unphased HLA alleles (DQA1*02:*01*, DQA1*05:*01*, DQA1*05:*05*, DQA1*03:*01*, DQB1*02:*01*, DQB1*02:*02*, DQB1*03:*01*, and DQB*03:*02*), we utilized HIBAG [49]. The quality of the imputed HLA alleles were measured using the INFO metric, INFO = Var(*x*) / (2 × *p* × (1 –*p*)) where *x* is the vector of imputed dosage for each allele and *p* is the imputed minor allele frequency, both estimated in controls only). The median INFO over the eight alleles was above 0.92 in all cohorts (**S5 Table)** indicating that the alleles were imputed well.

To evaluate whether each VIP was also independent of known CD risk haplotypes, we employed the likelihood ratio test, comparing two logistic regression models: (i) a logistic regression of the phenotype on the eight risk alleles and (ii) a logistic regression including both the HLA alleles and the VIP. The haplotypes were encoded as 8 allele dosages [53] and the VIP was encoded as 8 binary indicator variables. We considered an FDR threshold < 0.05, equivalent to *p* < 0.044, as statistically significant, indicating that adding the VIP to the model increased goodness-of-fit over the haplotypes alone (4,744 of the 5,454 tests were FDR-significant).

In addition, we used a logistic regression-based test for interactions, conditioning on known HLA haplotypes. We compared two logistic regression models: (i) the marginal SNPs and their interaction and (ii) the marginal SNPs alone. As interactions can be induced if two SNPs are in partial linkage with strong univariately-associated SNPs [43], both models were conditioned on the top five principal components, 8 known risk haplotypes and the top three associated SNPs found after conditioning on known risk haplotypes (*rs3129763 [hg18*.*chr6*:*g*.*32698903T>C]*, *rs2187668 [hg18*.*chr6*:*g*.*32713862T>C]* and, *rs3099844 [hg18*.*chr6*:*g*.*31556955T>G]*). Again, the haplotypes were encoded using a dosage encoding and VIPs were encoded as 8 binary indicator variables, while the associated individual SNPs were encoded using 2 binary indicator variables. This regression-based test for interaction is different to the model-free GSS statistic as it will only be able to capture a subset of possible interactions due to scale dependencies [62]. We used meta-analysis (Fisher’s method) to determine significance across the five CD cohorts. This is calculated as $X^{2}=-2\sum_{i=1}^{k}\ln(p_{i})$, where *k* is the number of independent studies (*k* = 5) and *p*_*i*_ is the significance of the LRT each of the cohorts. *X*^2^ is chi-squared distributed with 2*k* degrees of freedom and hence we can easily derive its significance. Using this meta-analysis approach, 521 VIPs were significant (after Bonferroni correction *P* = 9.12 × 10^−6^ = 0.05/5454) across the CD cohorts.

To confirm that the significance of interactions detected by this logistic-regression based test for interaction are not inflated by the presence of loci with large univariate effect size, we have simulated 100,000 sets of interactions under the null model of no significant interaction with and without a large univariate effect loci (defined as a locus having a univariate *X*^*2*^ significance below 10^−10^). Resulting Q-Q plots (**S7 Fig**) show no significant inflation for models with a strong loci compared to those without. Hence, the use of a logistic regression based test for interaction is unlikely to be highly skewed by loci of large effect.

### Estimating the number of independent interaction signals

To determine the number of independent signals coming from the VIPs, we used an LD pruning based approach to filter out all SNP pairs that are in disequilibrium with each other. To ensure that the interaction signals were not caused by haplotype effects, only SNP pairs that were deemed to be independent of HLA haplotypes were examined. Traditional measures of LD (such as *r*^2^ or *D’*) are designed for examining two binary loci whose frequencies can be reduced to a 2 × 2 table, whereas examining pairs of SNP pairs requires us to examine two multi-allelic loci, whose frequencies are naturally summarized by a 4 × 4 table. A widely used method dealing with this issue is the Hill's *Q* statistic, a multi-allelic extension of *r*^2^ [44]. It is well known that the *r*^2^ can be derived from the chi-squared test of association, since

$$r^{2}=\frac{1}{2n}\chi^{2}$$

where *n* is the total number of samples and *χ*^2^ is the chi-squared statistic over the haplotypes formed by the two SNPs. Motivated by this relationship, the *Q* statistic can be expressed as

$$Q=\frac{1}{2n}\sum_{i=1}^{k}\sum_{j=1}^{l}\frac{{\left(O_{ij}-E_{ij}\right)}^{2}}{E_{ij}}=\frac{1}{2n}\chi^{2}$$

where *O*_*ij*_ and *E*_*ij*_ are the observed and expected haplotype counts when there are *k* and *l* alleles respectively at the two loci. In the case of examining LD between pairs of SNP pairs *k* = *l* = 4. Phase information was inferred using SHAPEIT [63], and LD was then computed directly on control samples only. While it is somewhat arbitrary what threshold constitutes independence, given the direct analogy between *r*^2^ and *Q* we utilized a more conservative threshold of *Q* ≤ 0.3 than that commonly used for LD-pruning and tagging procedures (*r*^2^ ≤ 0.5), for example in PLINK. Such conservative thresholds may filter slightly less independent but still informative interaction signals, thus for the predictive models discussed below, all VIPs were initially allowed to enter the model. An exploration of different *Q* thresholds using different levels of population structure correction reveals that the number of independent signals is insensitive to the number of principal components included (**S8 Fig)**.

### The predictive models

We have employed a sparse support vector machine (SVM) implemented in SparSNP [64]. This is a multivariable linear model where the degree of sparsity (number of variables being assigned a non-zero weight) is tuned via penalization. The model is induced by minimizing the L1-penalized squared hinge loss

$$(\beta^{*},\beta_{0}^{*})=\arg\;\underset{\beta ,\beta_{0}}{\min}\;\frac{1}{2N}\sum_{i=1}^{N}\max{\left\{0,1-y_{i}(x_{i}^{T}\beta +\beta_{0})\right\}}^{2}+\lambda \sum_{j=1}^{p}\left|\beta_{j}\right|$$

where β and β_0_ are the model weights and the intercept, respectively, *N* is the number of samples, *p* is the number of variables (SNPs and/or encoded pairs), *x*_*i*_ is the *i*th vector of *p* variables (genotypes and/or encoded pairs), *y*_*i*_ is the *i*th case/control status {+1, −1}, and λ ≥ 0 is the L1 penalty. To find the optimal penalty, we used a grid of 100 penalty values within 10 replications of 10-fold cross-validation, and found the model/s that maximized the average area under the receiver-operating characteristic curve (AUC). For models based on single SNPs, we used minor allele dosage {0, 1, 2} encoding of the genotypes. For models based on VIPs, the standard dosage model is not applicable; hence, we transformed the variable representing each pair (encoded by integers 1 to 9) to 9 indicator variables, using a consistent encoding scheme across all datasets. The indicator variables were then analyzed in the same way as single SNPs.

### Evaluation of predictive ability and explained disease variance

To maximize the number of SNPs available for analysis, we imputed SNPs in the UK2, FIN, NL, and IT datasets to match those that were in the UK1 dataset but not in former, using IMPUTE v2.3.0 [65]. Imputed dosages were converted to PLINK hard calls (based on a minimum call probability threshold of 0.9). Within each dataset, hard-call SNPs were further filtered by MAF >1% and genotyping missingness <10%, and individuals filtered by <5% missingness. Post QC this left 290,277 SNPs common to all five datasets. Together with 9×5,454 validated interaction pairs = 49,086 indicator variables, this led to a total of 339,363 markers in the combined singles+VIP dataset. Models trained in cross-validation on the UK1 dataset were then applied without any further tuning to the four other datasets, and the external-validation AUC for these models was then estimated within the validation datasets. To derive the proportion of phenotypic variance explained by the model (on the liability scale), we used the method of Wray et al. [66] assuming a population prevalence of 1%.

## Supporting information

S1 Table Independent validated interaction signals detected in UK1 a)GSS indicates the–log_10_(p-value) of improvement of the pair over each of the SNPs involved measured by the adjusted GSS filter described further in the Methods section.b)Odds Ratios are calculated directly from the GSS rather than via logistic regression, discussed further in Methods.c)Minor Allele Frequency measured in the Control samples in the UK1 cohort.d)r^2^ between SNPs constituting the interaction.e)SNP positions were extracted from build 36.f)x^2^ indicates log10(p-value) for the standard x^2^ test of association (x^2^ statistics with 2 degrees of freedom).
(DOCX)

S2 Table Number of independent signals found across the different cohorts and their overlap. Overlap here is defined as the observing at least one pair in LD with the independent signal showing GSS significance across the two cohorts.
(DOCX)

S3 Table Genes regulated by eQTL SNPs from Table 3. eQTL data was collated from GTEX and RegulomeDB, and the unique set gene names are presented above.
(DOCX)

S4 Table Predictive power and disease variance explained by models with additive and interacting genetic effects, trained on a combined UK1 + UK2 dataset. Predictive power of single SNPS and pairs in cross-validation and in external validation, using SparSNP models. Models were optimized on the combined UK1 + UK2 dataset (n = 7,786 samples) in cross-validation (290K SNPs), and tested without modification on the other datasets. The 5,454 pairs were based on the UK1 dataset. The proportion of disease variance explained assumes a population prevalence of 1%. The 95% CI for AUC in UK1+UK2 was computed over the 10×10 cross-validation, and in external validation was computed using DeLong’s method (R package pROC).
(DOCX)

S5 Table HIBAG imputation quality within each celiac disease dataset. INFO and MAF were computed using the controls only, within each dataset. INFO = var(*x*) / (2 × *p* × (1 –*p*)), where *x* is the *n*-vector of imputed allele dosages (weighted by imputation posterior probability) and *p* is the MAF from the hard-called imputed alleles.
(DOCX)

S1 Fig Study workflow (PDF)

S2 Fig QQ plots for the GSS statistic in all datasets. We plot the distribution of–log_10_(p-values) from an exhaustive GSS scan against a uniform distribution expected under the null hypothesis of no interaction, using observed and permuted sample labels in blue and green respectively. In both cases, we observe a deviation from uniformity such that GSS p-values are liberal at P>10^−5^ but conservative at P<10^−5^. After applying the adjustment procedure (**S1 Text**), the resulting distribution of p-values exhibited no test statistic inflation (lambda = 1.00). Using either adjusted or unadjusted GSS values showed far lower p-values (capped at 10^−15^) than expected under permuted labels.
(PDF)

S3 Fig Cumulative distribution of LD between interacting pairs in the UK1 cohort pairs. LD was measured by phasing the data using SHAPEIT [63], and calculating r^2^ (top) and D’ (bottom) directly on control samples only
(TIF)

S4 Fig Correlation between GSS Significance and LD between interacting pairs in the UK1 cohort pairs. LD was measured by phasing the data using SHAPEIT [52], and calculating r^2^ (top) on control samples only.
(PDF)

S5 Fig Manhattan plots of the MHC for association of single SNPs (top panel) compared to association of interaction pairs (bottom panel). The top panel shows the strength of association with celiac disease in the UK1 dataset using the -log10(P) from a chi-squared test. The bottom panel shows the interaction effect of pairs which achieved Bonferroni-adjusted significant according to the GSS statistic. For each pair, we plot two points showing the location of the two constituent SNPs. The SNPs in the top 25 strongest pairs have been marked in orange in both plots. Vertical green and grey lines indicate selected genes with the width denoting gene size.
(PDF)

S6 Fig Manhattan plots of VIPs under a logistic regression based analysis conditional on known risk factors from a meta-analysis of all cohorts. We show *p*-values for all VIPs, with Bonferroni correction shown as a dashed line. 521 VIPs found to be significant past Bonferroni correction.
(PDF)

S7 Fig QQ plots comparing the distribution of significance for interactions where one loci has a strong univariate effect size to interactions where both loci are randomly generated. Using the SNPs from the MHC region of the UK1 cohort, we have repeatedly explored two different scenarios. In the first scenario (“Strong+Random” shown in red), we pair a locus of large effect (X^2^ p-value < 10^−10^) with a locus that has been simulated under the null hypothesis of no effect (achieved by permuting all genotypes across cases and controls). In the second scenario (“Random+Random” shown in blue), we utilize two loci simulated under the null hypothesis of no effect (again achieved by permuting all genotypes across cases and controls). Minor allele frequencies for the loci were determined by randomly sampling SNPs from the UK1 celiac cohort. For each scenario, we simulated 1 million pairs and generated a QQ plot of the results, shown above. Both scenarios show minimal deviation from a purely uniform distribution, indicating that the p-values are properly calibrated. Hence, the use of a logistic regression based test for interaction is unlikely to be highly skewed by loci of large effect.
(TIF)

S8 Fig Number of independent signals found using our LD pruning approaching as the *Q* statistic used to calculate multi-locus LD is varied. Additionally, we have examined the number of signals under different levels of population structure correction, including 0, 5 or 10 principal components in our regression model to test for haplotype independence.
(TIF)

S1 Text Supplementary Methods. (DOCX)

S1 Dataset 5,454 Validated Interacting Pairs and related statistics across the five celiac cohorts examined in this study. (XLS)
