# Supplementary material for: Interactions within the MHC contribute to the genetic architecture of celiac disease
Source: PLoS One. 2017 Mar 10;12(3):e0172826. doi: 10.1371/journal.pone.0172826 (PMC5345796; doi:10.1371/journal.pone.0172826)
Supplement: S1 Table — a)GSS indicates the–log10(p-value) of improvement of the pair over each of the SNPs involved measured by the adjusted GSS filter described further in the Methods section.b)Odds Ratios are calculated directly from the GSS rather than via logistic regression, discussed further in Methods.c)Minor Allele Frequency measured in the Control samples in the UK1 cohort.d)r2 between SNPs constituting the interaction.e)SNP positions were extracted from build 36.f)x2 indicates log10(p-value) for the standard x2 test of association (x2 statistics with 2 degrees of freedom). (DOCX) [file pone.0172826.s001.docx]

|  |  |  |  |  |  | UK1_univariate_ | | UK1 |  | | UK2 | | FIN | | NL | | IT | |
| --- | --- | --- | --- | --- | --- | --- | --- | --- | --- | --- | --- | --- | --- | --- | --- | --- | --- | --- |
|  | SNP | RS | Chr | Position (bp)^e^ | r^2 d^ | MAF^c^ | $X$^2 f^ | GSS^a^ | | OR^b^ | GSS^a^ | OR^b^ | GSS^a^ | OR^b^ | GSS^a^ | OR^b^ | GSS^a^ | OR^b^ |
| 1 | hg18.chr6:g.31701455G>A | rs2260000 | 6 | 31701455 | 0.34 | 0.28 | 40.86 | 58.29 | | 14.23 | 109.23 | 8.66 | 95.52 | 20.58 | 27.43 | 10.13 | 13.11 | 6.77 |
|  | hg18.chr6:g.31736712C>T | rs805262 | 6 | 31736712 |  | 0.47 | 24.67 |  |  |  |  |  |  |  |  |  |  |  |
| 2 | hg18.chr6:g.32777745G>A | rs2647050 | 6 | 32777745 | 0.32 | 0.32 | 16.3 | 28.99 | | 7.59 | 56.19 | 6.03 | 7.37 | 4.29 | 16.73 | 4.27 | 13.34 | 13.8 |
|  | hg18.chr6:g.32778934A>G | rs2856705 | 6 | 32778934 |  | 0.13 | 14.65 |  | |  |  |  |  |  |  |  |  |  |
| 3 | hg18.chr6:g.29719410A>G | rs29232 | 6 | 29719410 | 0.22 | 0.33 | 7.69 | 26.14 | | 4.22 | 39.16 | 3.63 | 32.92 | 6.64 | 18.26 | 4.69 | 3.68 | 7.44 |
|  | hg18.chr6:g.29775252T>C | rs7776082 | 6 | 29775252 |  | 0.5 | 14.68 |  | |  |  |  |  |  |  |  |  |  |
| 4 | hg18.chr6:g.32685358G>A | rs660895 | 6 | 32685358 | 0.01 | 0.18 | 30.11 | 19.45 | | 4.24 | 36.44 | 4.34 | 9.45 | 3.72 | 7.48 | 3.68 | 1.9 | 2.57 |
|  | hg18.chr6:g.32877641C>T | rs2219893 | 6 | 32877641 |  | 0.24 | 25.36 |  | |  |  |  |  |  |  |  |  |  |
| 5 | hg18.chr6:g.31192414T>C | rs1062470 | 6 | 31192414 | 0.06 | 0.29 | 12.48 | 18.96 | | 4.81 | 20.83 | 3.53 | 16.26 | 5.44 | 5.88 | 3.62 | 0.1 | 1.71 |
|  | hg18.chr6:g.31317489G>A | rs3130712 | 6 | 31317489 |  | 0.33 | 17.52 |  | |  |  |  |  |  |  |  |  |  |
| 6 | hg18.chr6:g.32259421T>C | rs2070600 | 6 | 32259421 | 0.02 | 0.05 | 15.08 | 16.8 | | 10.97 | 24.97 | 6 | 5.18 | 6.02 | 2.74 | 5.38 | 0.01 | 1.16 |
|  | hg18.chr6:g.32514320A>C | rs3129871 | 6 | 32514320 |  | 0.31 | 18.43 |  | |  |  |  |  |  |  |  |  |  |
| 7 | hg18.chr6:g.31429190T>C | rs2596501 | 6 | 31429190 | 0.05 | 0.42 | 25.45 | 16.11 | | 5.86 | 31.27 | 5.16 | 5.91 | 4.46 | 11.01 | 5.38 | 2.32 | 2.91 |
|  | hg18.chr6:g.31886251C>T | rs2227956 | 6 | 31886251 |  | 0.15 | 14.8 |  | |  |  |  |  |  |  |  |  |  |
| 8 | hg18.chr6:g.32484449C>A | rs3763313 | 6 | 32484449 | 0.06 | 0.14 | 21.98 | 14.99 | | 4.19 | 25.69 | 4.21 | 16.43 | 8.62 | 6.86 | 2.54 | 1.09 | 1.96 |
|  | hg18.chr6:g.32871088G>A | rs2621377 | 6 | 32871088 |  | 0.42 | 15.29 |  | |  |  |  |  |  |  |  |  |  |
| 9 | hg18.chr6:g.27520365A>G | rs7772160 | 6 | 27520365 | 0.31 | 0.48 | 6.96 | 13.7 | | 2.75 | 17.88 | 2.39 | 11.47 | 2.89 | 10.26 | 2.57 | 3.29 | 1.96 |
|  | hg18.chr6:g.27588896C>T | rs6918131 | 6 | 27588896 |  | 0.26 | 4.35 |  | |  |  |  |  |  |  |  |  |  |
| 10 | hg18.chr6:g.31630648A>G | rs6929796 | 6 | 31630648 | 0.08 | 0.14 | 18.57 | 13.52 | | 4.31 | 20.66 | 3.28 | 9.2 | 2.58 | 6.64 | 2.56 | 1.88 | 1.77 |
|  | hg18.chr6:g.31644203T>C | rs2844484 | 6 | 31644203 |  | 0.34 | 17.01 |  | |  |  |  |  |  |  |  |  |  |
| 11 | hg18.chr6:g.33147603C>T | rs9277341 | 6 | 33147603 | 0.26 | 0.35 | 11.43 | 13.54 | | 3.5 | 19.79 | 2.76 | 25.17 | 5.01 | 14.45 | 3.61 | 3.15 | 2.26 |
|  | hg18.chr6:g.33191099G>A | rs1810472 | 6 | 33191099 |  | 0.27 | 4.94 |  |  |  |  |  |  |  |  |  |  |  |
| 12 | hg18.chr6:g.32919361G>A | rs6924102 | 6 | 32919361 | 0.13 | 0.5 | 11.46 | 13.39 | | 3.06 | 16.23 | 3.01 | 8.95 | 4.16 | 6.64 | 3.16 | 0.44 | 1.44 |
|  | hg18.chr6:g.32919607A>C | rs2071543 | 6 | 32919607 |  | 0.12 | 10.74 |  | |  |  |  |  |  |  |  |  |  |
| 13 | hg18.chr6:g.31572718T>C | rs3828903 | 6 | 31572718 | 0 | 0.27 | 21.04 | 12.25 | | 3.35 | 18.18 | 3 | 9.85 | 4.13 | 7.51 | 3.13 | 2.44 | 2.74 |
|  | hg18.chr6:g.32845485A>G | rs9368741 | 6 | 32845485 |  | 0.21 | 25.11 |  | |  |  |  |  |  |  |  |  |  |
| 14 | hg18.chr6:g.31002738T>C | rs2532934 | 6 | 31002738 | 0.01 | 0.43 | 32.95 | 12.28 | | 5 | 15.9 | 4.13 | 9.62 | 4.92 | 9.38 | 5.86 | 2.31 | 3.17 |
|  | hg18.chr6:g.32517508G>A | rs3129882 | 6 | 32517508 |  | 0.33 | 35.38 |  | |  |  |  |  |  |  |  |  |  |

**S1 Table. Independent validated interaction signals detected in UK1.**

1. GSS indicates the –log_10_(p-value) of improvement of the pair over each of the SNPs involved measured by the adjusted GSS filter described further in the Methods section .
2. Odds Ratios are calculated directly from the GSS rather than via logistic regression, discussed further in Methods.
3. Minor Allele Frequency measured in the Control samples in the UK1 cohort.
4. r^2^ between SNPs constituting the interaction.
5. SNP positions were extracted from build 36.
6. $X^{2}$ indicates log10(p-value) for the standard $\chi$^2^ test of association ($\chi$^2^ statistics with 2 degrees of freedom).
