# Supplementary material for: Interactions within the MHC contribute to the genetic architecture of celiac disease
Source: PLoS One. 2017 Mar 10;12(3):e0172826. doi: 10.1371/journal.pone.0172826 (PMC5345796; doi:10.1371/journal.pone.0172826)
Supplement: S2 Table — Overlap here is defined as the observing at least one pair in LD with the independent signal showing GSS significance across the two cohorts. (DOCX) [file pone.0172826.s002.docx]

| **Discovery**  **dataset** | **# of replicating  SNP pairs** | **# of indep  signals** | **# of replicating signals** | | | | | |
| --- | --- | --- | --- | --- | --- | --- | --- | --- |
|  |  |  | **UK1** | **UK2** | **FIN** | **NL** | **IT** |  |
| UK1 | 5,454 | 14 | - | 14 | 8 | 3 | 2 |  |
| UK2 | 30,351 | 17 | 16 | - | 16 | 5 | 3 |  |
| FIN | 8,058 | 7 | 5 | 7 | - | 3 | 1 |  |
| NL | 819 | 4 | 3 | 4 | 3 | - | 2 |  |
| IT | 397 | 1 | 1 | 1 | 1 | 1 | - |  |

**S2 Table. Number of independent signals found using the different cohorts for discovery and the replication of these signals across the remaining cohorts.** For each dataset**,** we have applied an LD pruning approach to the set of GSS significant SNP pairs that were shown to replicate across cohorts (see “Estimating the number of independent interaction signals in main paper”). For each signal found in a given discovery cohort, we have recorded the number of signals that replicate across cohorts where replication is defined as finding at least one SNP pair from the independent signal that is also haplotype independent in the replication cohort. Note that the number of signals replicated in a cohort may not reflect the number of independent signals found in that cohort (e.g. there are 16 UK2 signals shown to replicate in UK1 but only 14 independent signals discovered in UK1). This is a result of changes in LD across cohorts leading to different pruning results and hence altering the number of detected signals.
