# Supplementary material for: Interactions within the MHC contribute to the genetic architecture of celiac disease
Source: PLoS One. 2017 Mar 10;12(3):e0172826. doi: 10.1371/journal.pone.0172826 (PMC5345796; doi:10.1371/journal.pone.0172826)
Supplement: S3 Table — eQTL data was collated from GTEX and RegulomeDB, and the unique set gene names are presented above. (DOCX) [file pone.0172826.s003.docx]

| **eQTL** | **Rs ID** | **Gene** |
| --- | --- | --- |
| hg18.chr6:g.31192414T>C | rs1062470 | C6orf15, CCHCR1, CDSN, CYP21A1P, HCG21, HCG22, HCG27, HLA-C, HLA-L, LINC00243, MICB, POU5F1, PSORS1C1, PSORS1C2, PSORS1C3, TCF19, XXbac-BPG181B23.7, XXbac-BPG248L24.12, XXbac-BPG299F13.17 |
| hg18.chr6:g.32919607A>C | rs2071543 | TAP1, TAP2 |
| hg18.chr6:g.31429190T>C | rs2596501 | CCHCR1, HCG22, HCG27, HLA-B, HLA-C, MICA, POU5F1, PRRT1, XXbac-BPG181B23.7, XXbac-BPG299F13.17 |
| hg18.chr6:g.31644203T>C | rs2844484 | ATP6V1G2, C4A, CCHCR1, DDX39B, HCP5, HLA-DRB5, MICB, XXbac-BPG181B23.7, HLA-C, LST1, NA, BAT1 |
| hg18.chr6:g.31572718T>C | rs3828903 | HLA-B, HLA-C, LY6G5B, MICB, PRRT1 |
| hg18.chr6:g.32685358G>A | rs660895 | HLA-DOB, HLA-DQA1, HLA-DQA2, HLA-DQB1, HLA-DQB1-AS1, HLA-DQB2, HLA-DRB1, HLA-DRB6, LY6G5B, XXbac-BPG254F23.6 |
| hg18.chr6:g.27588896C>T | rs6918131 | RP1-153G14.4, ZNF184, ZNF204P, ZNF391 |
| hg18.chr6:g.32919361G>A | rs6924102 | PSMB9, TAP1, TAP2 |
| hg18.chr6:g.31630648A>G | rs6929796 | CYP21A1P, DDX39B, HCG20, HLA-C, MICB |
| hg18.chr6:g.27520365A>G | rs7772160 | OR2B8P, RP1-153G14.4, ZNF184, ZNF192P1, ZNF204P, ZNF391 |
| hg18.chr6:g.31317489G>A | rs3130712 | CCHCR1 |
| hg18.chr6:g.31886251C>T | rs2227956 | HLA-DRB1, HLA-C, HLA-DQB1, HLA-DRB5 |
| hg18.chr6:g.32484449C>A | rs3763313 | HLA-DQA1, HLA-DRB5 |
| hg18.chr6:g.33147603C>T | rs9277341 | HLA-DPB1 |
| hg18.chr6:g.33191099G>A | rs1810472 | B3GALT4, GPSM3, PBX2, AY090769, RPS18, HLA-DMA, TAPBP |
| hg18.chr6:g.31002738T>C | rs2532934 | HLA-C |
| hg18.chr6:g.32517508G>A | rs3129882 | HLA-DQA2, HLA-DRA, HLA-DRB5 |

**S3 Table. Genes regulated by eQTL SNPs from Table 3.** eQTL data was collated from GTEX and RegulomeDB, and the unique set gene names are presented above.
