# Supplementary material for: Interactions within the MHC contribute to the genetic architecture of celiac disease
Source: PLoS One. 2017 Mar 10;12(3):e0172826. doi: 10.1371/journal.pone.0172826 (PMC5345796; doi:10.1371/journal.pone.0172826)
Supplement: S4 Table — Predictive power of single SNPS and pairs in cross-validation and in external validation, using SparSNP models. Models were optimized on the combined UK1 + UK2 dataset (n = 7,786 samples) in cross-validation (290K SNPs), and tested without modification on the other datasets. The 5,454 pairs were based on the UK1 dataset. The proportion of disease variance explained assumes a population prevalence of 1%. The 95% CI for AUC in UK1+UK2 was computed over the 10×10 cross-validation, and in external validation was computed using DeLong’s method (R package pROC). (DOCX) [file pone.0172826.s004.docx]

|  |  | **Single SNPs** | | **Combined (single SNPs + pairs)** | | **Validated interaction pairs** | |
| --- | --- | --- | --- | --- | --- | --- | --- |
|  |  | **Var. Exp.** | **AUC (95% CI)** | **Var. Exp.** | **AUC (95% CI)** | **Var. Exp.** | **AUC (95% CI)** |
| Cross validation | UK1+UK2 | 0.320 | 0.879 [0.878, 0.879] | 0.335 | 0.885 [0.885, 0.886] | 0.317 | 0.878 [0.877, 0.878] |
| External validation | Finn | 0.353 | 0.892 [0.879, 0.906] | 0.368 | 0.898 [0.885, 0.911] | 0.347 | 0.890 [0.876, 0.904] |
|  | IT | 0.288 | 0.864 [0.843, 0.886] | 0.309 | 0.874 [0.853, 0.895] | 0.288 | 0.864 [0.842, 0.887] |
|  | NL | 0.298 | 0.869 [0.852, 0.886] | 0.298 | 0.869 [0.852, 0.886] | 0.291 | 0.866 [0.848, 0.884] |

**S4 Table. Predictive power and disease variance explained by models with additive and interacting genetic effects, trained on a combined UK1 + UK2 dataset.** Predictive power of single SNPS and pairs in cross-validation and in external validation, using SparSNP models. Models were optimized on the combined UK1 + UK2 dataset (n=7,786 samples) in cross-validation (290K SNPs), and tested without modification on the other datasets. The 5,454 pairs were based on the UK1 dataset. The proportion of disease variance explained assumes a population prevalence of 1%. The 95% CI for AUC in UK1+UK2 was computed over the 10×10 cross-validation, and in external validation was computed using DeLong’s method (R package pROC).
