# Supplementary material for: Interactions within the MHC contribute to the genetic architecture of celiac disease
Source: PLoS One. 2017 Mar 10;12(3):e0172826. doi: 10.1371/journal.pone.0172826 (PMC5345796; doi:10.1371/journal.pone.0172826)
Supplement: S5 Table — INFO and MAF were computed using the controls only, within each dataset. INFO = var(x) / (2 × p × (1 –p)), where x is the n-vector of imputed allele dosages (weighted by imputation posterior probability) and p is the MAF from the hard-called imputed alleles. (DOCX) [file pone.0172826.s005.docx]

|  | **UK1**  (1422 controls) | | **UK2**  (4936 controls) | | **NL**  (846 controls) | | **FIN**  (1829 controls) | | **IT**  (543 controls) | |
| --- | --- | --- | --- | --- | --- | --- | --- | --- | --- | --- |
| **Allele** | **INFO** | **MAF** | **INFO** | **MAF** | **INFO** | **MAF** | **INFO** | **MAF** | **INFO** | **MAF** |
| DQA1*02:01 | 0.957 | 0.143 | 0.977 | 0.149 | 1.001 | 0.103 | 0.986 | 0.062 | 0.939 | 0.122 |
| DQA1*05:01 | 1.011 | 0.138 | 0.100 | 0.143 | 0.969 | 0.154 | 0.965 | 0.095 | 0.972 | 0.085 |
| DQA1*05:05 | 0.885 | 0.093 | 0.947 | 0.093 | 0.959 | 0.105 | 0.968 | 0.086 | 0.102 | 0.299 |
| DQA1*03:01 | 0.605 | 0.114 | 0.855 | 0.103 | 0.792 | 0.104 | 0.787 | 0.125 | 0.836 | 0.048 |
| DQB1*02:01 | 0.980 | 0.138 | 0.999 | 0.143 | 0.964 | 0.154 | 0.963 | 0.095 | 0.957 | 0.085 |
| DQB1*02:02 | 0.983 | 0.101 | 0.995 | 0.107 | 0.934 | 0.072 | 0.949 | 0.049 | 0.887 | 0.101 |
| DQB1*03:01 | 0.730 | 0.188 | 0.950 | 0.181 | 0.951 | 0.151 | 0.941 | 0.119 | 1.015 | 0.325 |
| DQB1*03:02 | 0.703 | 0.115 | 0.985 | 0.105 | 0.944 | 0.108 | 0.958 | 0.125 | 0.955 | 0.054 |
| Median | 0.921 | - | 0.981 | - | 0.955 | - | 0.961 | - | 0.956 | - |

**Table S3:** HIBAG imputation quality within each celiac disease dataset. INFO and MAF were computed using the controls only, within each dataset. INFO = var(*x*) / (2 × *p* × (1 – *p*)), where *x* is the *n*-vector of imputed allele dosages (weighted by imputation posterior probability) and *p* is the MAF from the hard-called imputed alleles.
