# Supplementary figures and images for: Interactions within the MHC contribute to the genetic architecture of celiac disease
Source: PLoS One. 2017 Mar 10;12(3):e0172826. doi: 10.1371/journal.pone.0172826 (PMC5345796; doi:10.1371/journal.pone.0172826)

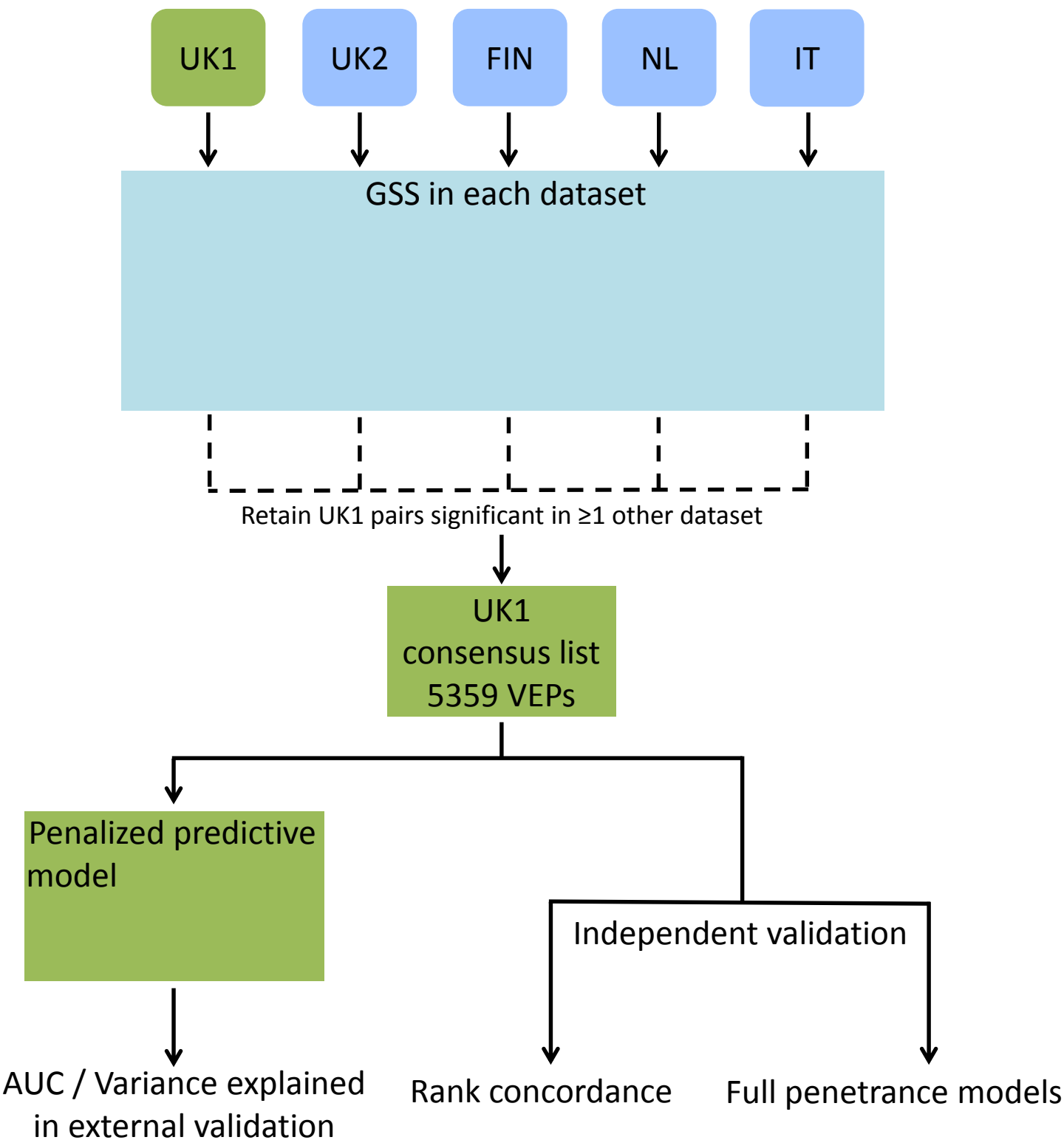

Supplement: S1 Fig — (PDF) [file pone.0172826.s006.pdf]

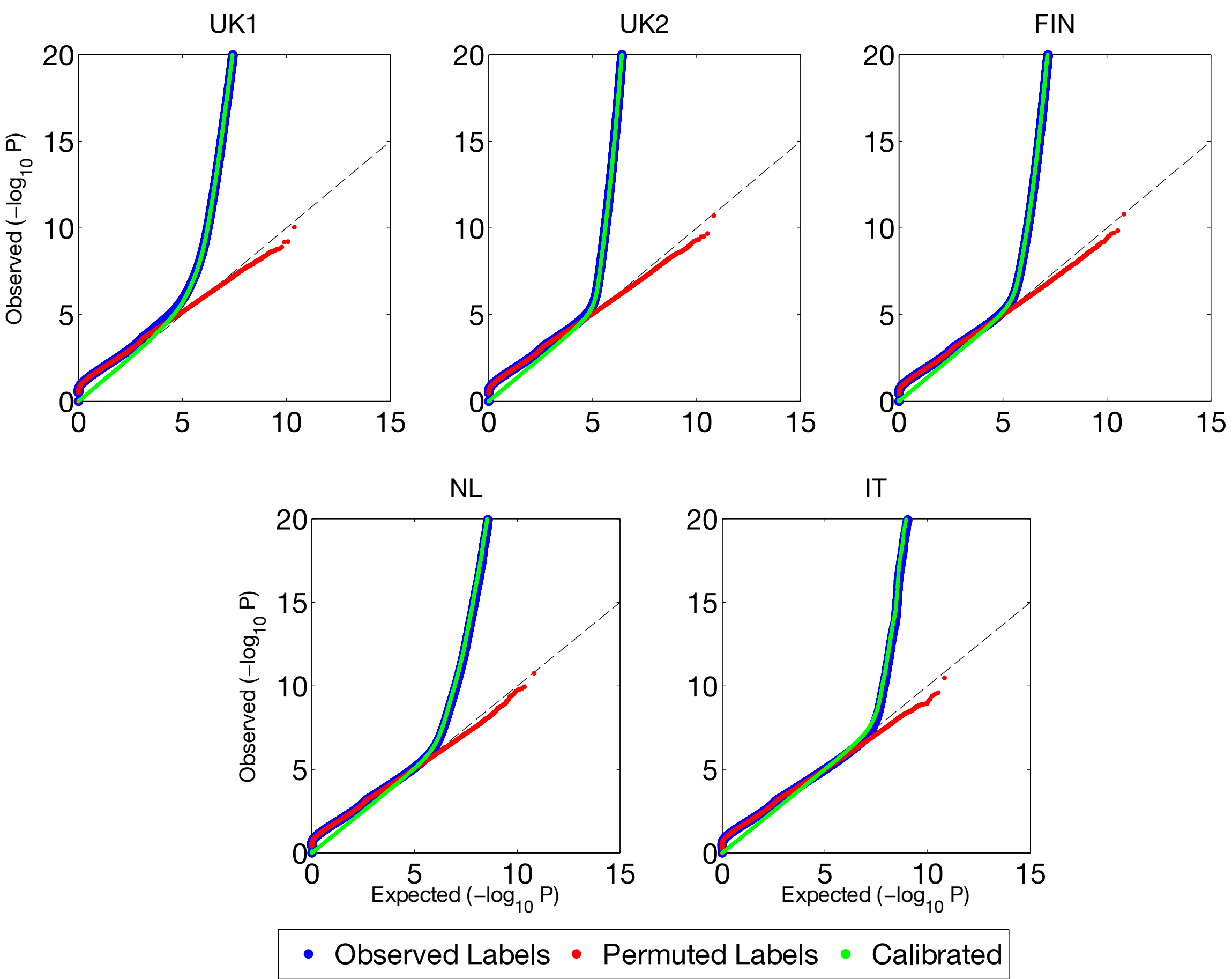

Supplement: S2 Fig — We plot the distribution of–log10(p-values) from an exhaustive GSS scan against a uniform distribution expected under the null hypothesis of no interaction, using observed and permuted sample labels in blue and green respectively. In both cases, we observe a deviation from uniformity such that GSS p-values are liberal at P>10−5 but conservative at P<10−5. After applying the adjustment procedure (S1 Text), the resulting distribution of p-values exhibited no test statistic inflation (lambda = 1.00). Using either adjusted or unadjusted GSS values showed far lower p-values (capped at 10−15) than expected under permuted labels. (PDF) [file pone.0172826.s007.pdf]

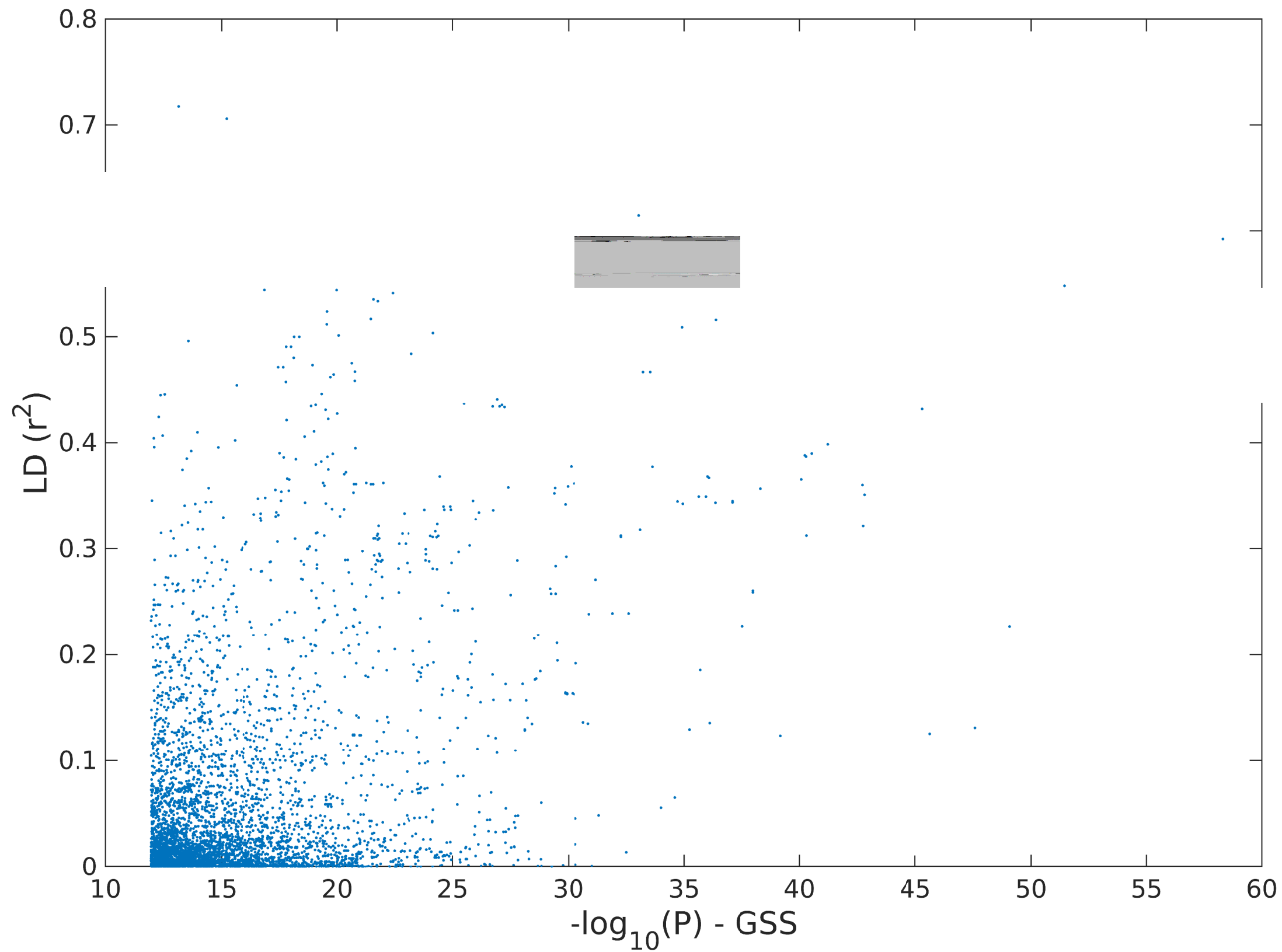

Supplement: S4 Fig — LD was measured by phasing the data using SHAPEIT [52], and calculating r2 (top) on control samples only. (PDF) [file pone.0172826.s009.pdf]

# celUK1 – Univariate $\chi^2$

$-\log_{10}(P): \text{Chi2}_{1w}$

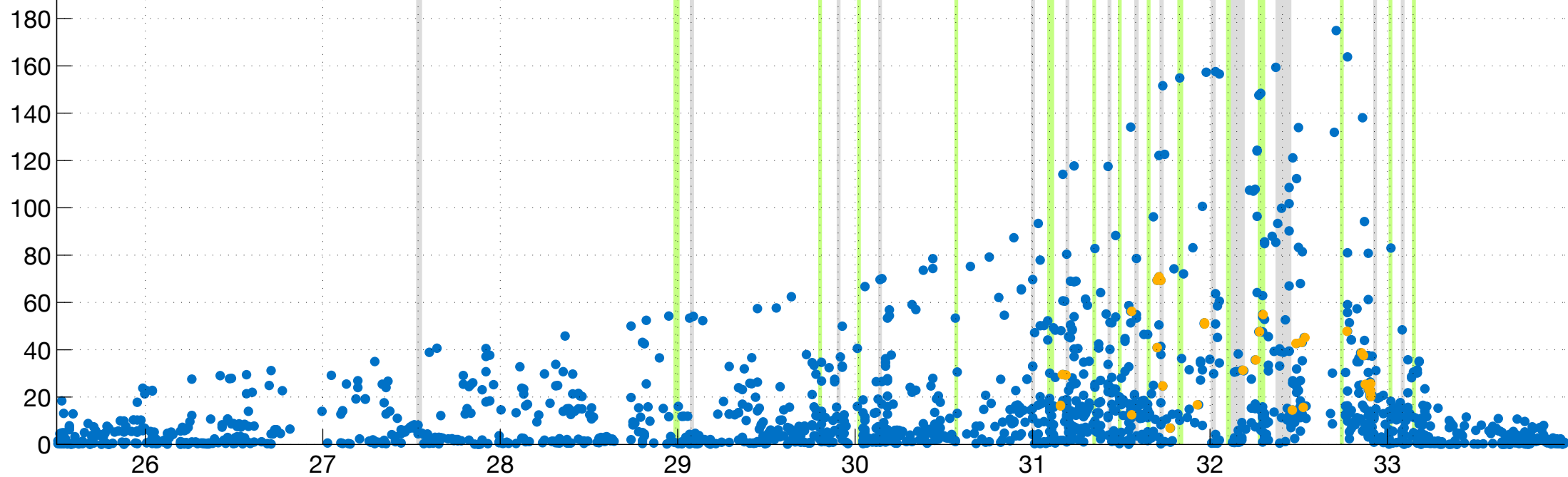

Pair – GSS

$-\log_{10}(P): \text{GSS}$

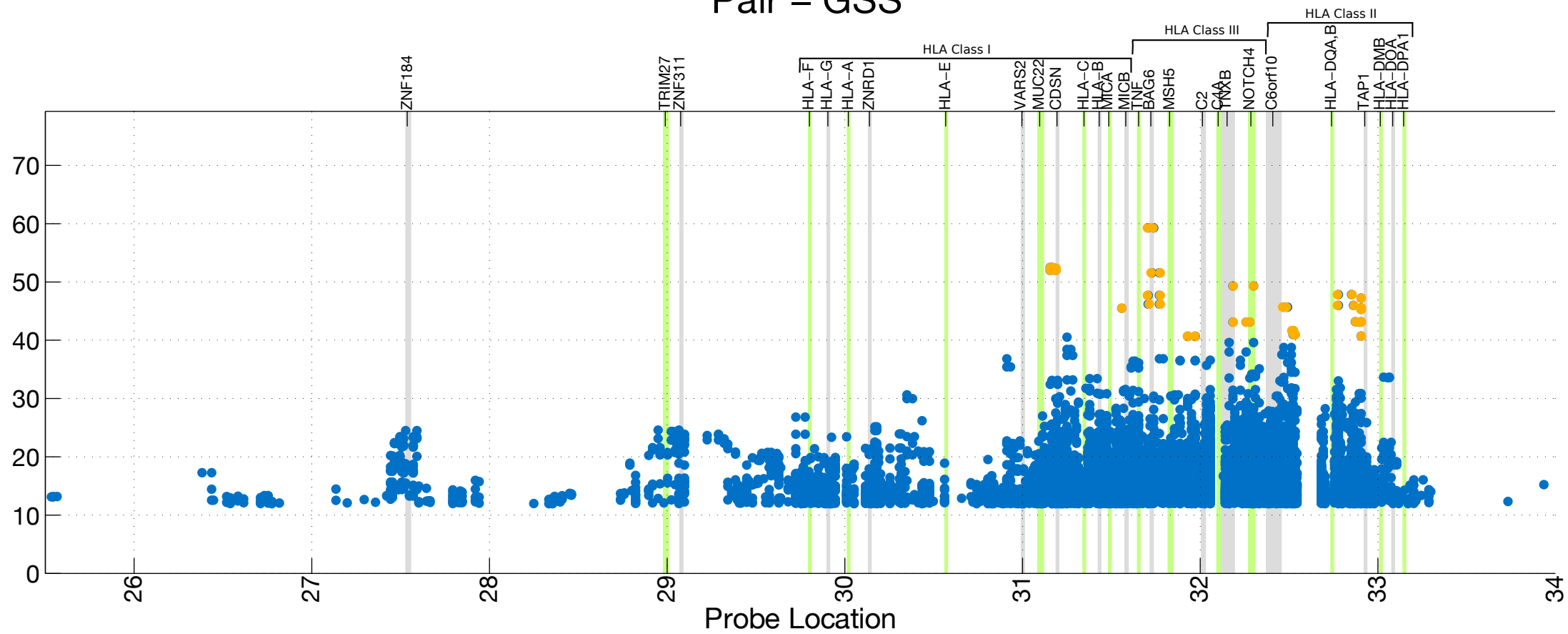

Supplement: S5 Fig — The top panel shows the strength of association with celiac disease in the UK1 dataset using the -log10(P) from a chi-squared test. The bottom panel shows the interaction effect of pairs which achieved Bonferroni-adjusted significant according to the GSS statistic. For each pair, we plot two points showing the location of the two constituent SNPs. The SNPs in the top 25 strongest pairs have been marked in orange in both plots. Vertical green and grey lines indicate selected genes with the width denoting gene size. (PDF) [file pone.0172826.s010.pdf]

# Meta-analysis (5 studies)

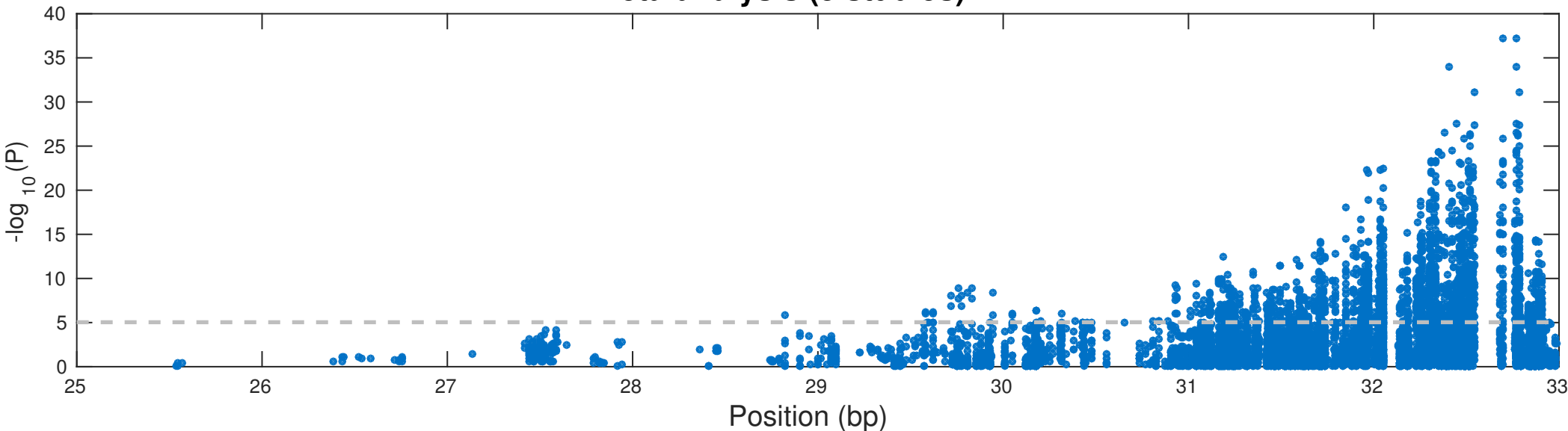

Supplement: S6 Fig — We show p-values for all VIPs, with Bonferroni correction shown as a dashed line. 521 VIPs found to be significant past Bonferroni correction. (PDF) [file pone.0172826.s011.pdf]

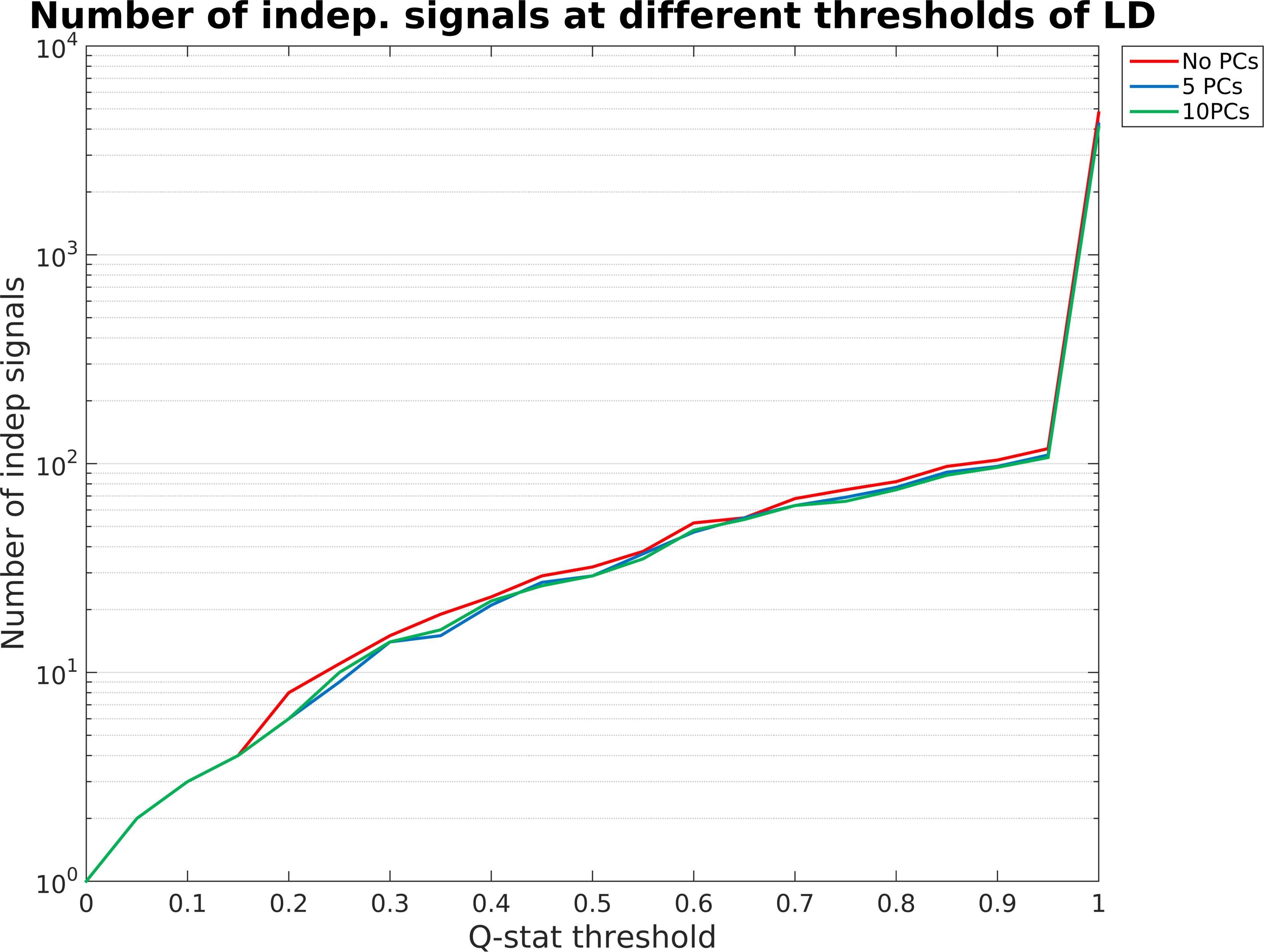

Supplement: S8 Fig — Additionally, we have examined the number of signals under different levels of population structure correction, including 0, 5 or 10 principal components in our regression model to test for haplotype independence. (TIF) [file pone.0172826.s013.tif]
