# Supplementary material for: Interactions within the MHC contribute to the genetic architecture of celiac disease
Source: PLoS One. 2017 Mar 10;12(3):e0172826. doi: 10.1371/journal.pone.0172826 (PMC5345796; doi:10.1371/journal.pone.0172826)
Supplement: S1 Text — (DOCX) [file pone.0172826.s014.docx]

**­­­­­Supplementary Materials**

**Interactions within the MHC contribute to the genetic architecture of celiac disease**

Benjamin Goudey^1,2,3^, Gad Abraham^4,5,6^, Eder Kikianty^7^, Qiao Wang^1,2^, Dave Rawlinson^1,2^, Fan Shi^1,2^, Izhak Haviv^8^, Linda Stern^2^, Adam Kowalczyk^1,2,9,*^, Michael Inouye^4,5,6,*^

^1^ NICTA Victoria Research Lab, The University of Melbourne, Parkville, Victoria 3010, Australia

^2^ Department of Computing and Information Systems, The University of Melbourne, Parkville, Victoria 3010, Australia

^3^ IBM Research, Australia, Level 5, 204 Lygon Street, Carlton, Victoria 3206, Australia

^4^ Centre for Systems Genomics, The University of Melbourne, Parkville, Victoria 3010, Australia

^5^ School of BioSciences, The University of Melbourne, Parkville, Victoria 3010, Australia

^6^ Department of Pathology, The University of Melbourne, Parkville, Victoria 3010, Australia

^7^ Department of Mathematics, University of Johannesburg, PO Box 524, Auckland Park 2006, South Africa

^8^ Faculty of Medicine, Bar Ilan University, Safed, Israel

^9^ Center for Neural Engineering, The University of Melbourne, Parkville, Victoria 3010, Australia

^*^These authors contributed equally

Correspondence should be addressed to Michael Inouye (minouye@unimelb.edu.au)

**Supplementary Text**

## *Statistical tests for interaction*

Here, we summarize the Gain in Sensitivity and Specificity (GSS) test employed to detect interactions. The test has been presented in detail in [[36](#_ENREF_36)] and is available at <https://github.com/bwgoudey/gwis-stats>.

There is a long history of discussion around the definition of epistasis, or gene-gene interaction [[39](#_ENREF_39)]. Here, an SNP interaction is defined as a significant improvement of a SNP-pair in classifying cases from controls over what is possible using each SNP individually. There are two main differences between our approach and regression-based approaches for detecting interactions [[8](#_ENREF_8),[26](#_ENREF_26)]. First, our approach is “model-free”, as it makes no assumptions about the way in which genotypes combine to affect the phenotype [[7](#_ENREF_7),[50](#_ENREF_50)], but considers all possible pairwise interactions for each pair, making it potentially more powerful to detect unknown interaction forms, as empirical knowledge about SNP interactions in humans is currently lacking. Second, instead of measuring the deviation from additive effects (for example, using a likelihood ratio test), our approach focuses on the utility of the test in case/control classification, quantified using the receiver-operating characteristic (ROC) curves.

The main principle behind the GSS is quantification of the gain in predictive power afforded by a putative interacting pair over and above the predictive power due to each of its constituent SNPs. The improvement in predictive power is assessed in terms of comparison of two curves; the ROC curve for the pair of SNPs (pair-ROC) and the convex hull of ROC curves formed by each of the two individual SNPs. This convex hull is, simplistically, the best ROC curve that can be produced by any convex combination of the predictions from two individual SNPs and represents a conservative estimate of the predictive power of the individual SNPs. Each of these ROC curves is equivalent to an optimal (Bayesian) classifier as no other classifier created from the given genotype data alone will outperform it. These ROC curves are formed by considering each possible genotype (or pair of genotypes) separately, then to sorting them according to the disease risk and using the sorted sequence to form a sequence of classifiers such that all samples carrying the genotypes up to the point of the ordered sequence are assigned to cases, while all others are assigned to controls. Using this, we then measure the sensitivity (true positive rate, TPR) and specificity (1 – false positive rate, FPR) and allocate the ratio TPR/FPR (i.e. likelihood ratio) to all samples carrying this genotype. After such ratios have created for all genotypes, and hence ratios allocated to all samples, a piecewise optimal ROC curve can be formed.

The improvement in the predictive power of the pair of SNPs is quantified by assessing the shape of those curves, rather than using the standard metric of the areas under them. More precisely, the GSS statistics assigns a p-value to each point in the pair-ROC curve, equal to the probability of observing a sample of genotypes with a higher or equal TPR and a lower or equal FPR, under the null hypothesis that the true TPR and FPR reside below the convex hull. We employ a highly efficient minimax-based implementation, maximizing the probability for each point on the ROC curve (worst case scenario) against all points of the convex hull, and returning the minimum probability over all points [[36](#_ENREF_36)]; this is done using an exact procedure rather than relying on approximations based on the normal distribution. Finally, the best p-value is assigned as the overall p-value for the pair, allowing the pairs to be ranked and corrected for multiple testing as is standard practice in GWAS. Those SNPs that are significant after multiple testing correction are deemed significantly interacting pairs.

## *Multiple testing burden*

Due to the vast number of SNP pairs being tested, a multiple testing adjustment must be made to control the false positive rate. Here, we utilize a simple Bonferroni correction for the number of unique SNP pairs tested: *P* = 1.1 x 10^-12^ for the UK1 and *P* = 3.75 x 10^-13^ for the other datasets. As with any adjustment, the cost of false positives versus that of false negatives must be weighed. We rationalize this selected simple threshold as a balance between competing the competing views that (a) a genome-wide scan for interactions should utilize a genome-wide significance threshold based on that used for univariate GWAS (e.g. *P* < (5x10^-8^)^2^ / 2), and (b) a false discovery rate approach (e.g. Benjamini-Hochberg). Since we observe wide concordance between independent datasets of two-locus models and interaction pair rank, it is likely that our threshold is not excessively permissive and/or is set appropriately, however, due to the historic difficulty in replicating interaction signals, we maintain our arguably conservative approach based on the number of unique tests and acknowledge that the consequences of various multiple testing adjustments in genome-wide interaction scans is still an open question. It is clear that further studies specifically addressing this are needed.

## *Adjustment for non-uniformity of p-values*

The GSS statistic showed non-uniformity in the data over the observed labels and after permutation of sample levels, indicating that the resulting distribution is a product of the statistical test rather than the data (**Figure S2**). To properly calibrate the GSS, we used an approach analogous to the widely accepted genomic control method [56]. Here, we permuted the sample labels to build an empirical null distribution which can be used to create an adjustment procedure for each observed p-value, which can be expressed as:

$P_{adj}=P_{obs}\times\frac{P_{uni}}{P_{perm}}$.

For a given observed p-value *P_obs_*, we determined the expected p-value under the uniform distribution, *P_uni_*, and the corresponding p-value observed in the permuted data *P_perm_* which has the same *P_uni_* expected under the null hypothesis of no interaction. We then formed a scaling factor $\frac{P_{uni}}{P_{perm}}$ to adjust the observed values in a manner analogous to lambda in genomic control. This adjustment procedure is used to normalise all GSS p-values. The adjusted GSS p-values displayed lambda values of 1.00, indicating no inflation relative to the uniform distribution (**Figure S2**). All p-values reported in this work have been adjusted by the proposed method.
